# Immune Profile and Risk of COVID-19-Associated Pulmonary Aspergillosis (CAPA): A Multicenter Retrospective Study with Matching Cohorts

**DOI:** 10.3390/jof12100763

**Published:** 2026-10-10

**Authors:** Olivier Mascle, Claire Dupuis, Antoine Lafarge, Maxime Moniot, Bertrand Evrard, Philippe Poirier, Adrien Joseph, Elie Azoulay, Guillaume Dumas, Thibault Dupont, Anne-Claire Lukaszewicz, the RICO Study Group, Salma Souilhi, Cécile Henquell, Fabienne Venet, Benjamin Bonnet, Bertrand Souweine

**Affiliations:** 1 Medical Intensive Care Unit, Gabriel-Montpied Hospital, CHU de Clermont-Ferrand, 63000 Clermont-Ferrand, France; cdupuis1@chu-clermontferrand.fr (C.D.); ssouilhi@chu-clermontferrand.fr (S.S.); bsouweine@chu-clermontferrand.fr (B.S.); 2 INRAE, Unité de Nutrition Humaine, Université Clermont Auvergne, CRNH Auvergne, 63000 Clermont-Ferrand, France; 3 Medical Intensive Care Unit, Hôpital Saint-Louis, 75010 Paris, France; antoine.lafarge@aphp.fr (A.L.); adrien.joseph@aphp.fr (A.J.); elie.azoulay@aphp.fr (E.A.); gdumasgalant@chu-grenoble.fr (G.D.);; 4 Service de Parasitologie-Mycologie, CHU Clermont-Ferrand, 3iHP, 63000 Clermont-Ferrand, France; mmoniot@chu-clermontferrand.fr (M.M.); ppoirier@chu-clermontferrand.fr (P.P.); 5 Service d’Immunologie, CHU Gabriel-Montpied, 63000 Clermont-Ferrand, France; bevrard@chu-clermontferrand.fr (B.E.); bbonnet@chu-clermontferrand.fr (B.B.); 6 Laboratoire d’Immunologie, ECREIN, UMR1019 UNH, UFR Médecine de Clermont-Ferrand, Université Clermont Auvergne, 63000 Clermont-Ferrand, France; 7 Joint Research Unit HCL-bioMérieux, EA 7426 “Pathophysiology of Injury-Induced Immunosuppression”, Hospices Civils de Lyon-bioMérieux, Université Claude Bernard Lyon 1, 69003 Lyon, France; anne-claire.lukaszewicz@chu-lyon.fr; 8 Anesthesia and Critical Care Medicine Department, Edouard Herriot Hospital, Hospices Civils de Lyon, 69437 Lyon, France; 9 Immunology Laboratory, Hôpital E. Herriot-Hospices Civils de Lyon, 5 place d’Arsonval, 69437 Lyon, Francefabienne.venet@chu-lyon.fr (F.V.); 10 Laboratoire de virologie, CHU de Clermont-Ferrand, 63000 Clermont-Ferrand, France; chenquell@chu-clermontferrand.fr; 11 Centre International de Recherche en Infectiologie (CIRI), Inserm U1111, CNRS, UMR5308, Ecole Normale Supérieure de Lyon, Université Claude, Bernard-Lyon 1, 69100 Villeurbanne, France; 12 Laboratoire Microorganismes, Génome et Environnement, Université Clermont Auvergne, CNRS, 63000 Clermont-Ferrand, France

**Keywords:** *Aspergillus*, CAPA, cytokines, COVID-19, immune profile

## Abstract

**Background**: The anti-inflammatory effects of COVID-19 and its treatment may promote COVID-19-associated pulmonary aspergillosis (CAPA). This study aimed to investigate the immunological profile associated with CAPA in ICU patients. **Methods**: We conducted a retrospective study in three French ICUs. Severe COVID-19 patients who underwent immunological profiling within 10 days of ICU admission were included. CAPA patients were matched with non-CAPA patients to assess risk factors. **Results**: Among 479 non-CAPA patients, 52 were matched with 28 CAPA patients. CAPA patients more frequently required organ support (*p* < 0.01), but mortality rates were similar. At ICU admission and day 7, CAPA patients had higher serum IL-6, IL-10, and IL-6/mHLA-DR ratios. In univariate conditional logistic regression an mHLA-DR expression > 8000 antibodies/cell at admission was associated with lower CAPA (OR = 0.41 [0.17–0.99], *p* = 0.04) and CAPA or death (OR = 0.26 [0.08–0.81], *p* = 0.04). An IL-6/mHLA-DR ratio > 2.8 tended to be associated with CAPA or death (OR = 3.16 [0.98–10.21], *p* = 0.05). Multivariate analysis did not find an association between immunological data and development of CAPA. **Conclusions**: We did not find an association between early immunological profiling data and development of CAPA in multivariate analysis. However, mHLA-DR, IL-10 and IL-6/mHLA-DR ratios may be interesting directions for future research with larger cohorts.

## 1. Introduction

In intensive care unit (ICU) patients, COVID-19-induced acute hypoxemic respiratory failure (COVID-AHRF) is characterized by immunological dysregulation combining both a proinflammatory response and immune paralyzing effects that lead to profound immunosuppression [1,2,3,4]. This dysregulation has been associated with viral dissemination, severe COVID-AHRF, increased risk of coinfections, and poorer outcomes [1,2,4]. COVID-19-associated invasive pulmonary aspergillosis (CAPA) [5] is one of the most serious coinfections during the COVID-19 pandemic [6] and is reportedly associated with a prolonged duration of invasive mechanical ventilation (IMV), longer ICU and hospital stays, and increased mortality [6,7]. Reported incidence of CAPA ranges between 2.5% and 47.2% in ICUs, mostly depending of mycological sampling policy [6,8,9,10,11].

A decrease in protective pulmonary immunity classically promotes the development of invasive pulmonary aspergillosis (IPA) [12,13,14,15,16,17]. In COVID-AHRF, several conditions contribute to both systemic and compartmental immunosuppression, potentially leading to a predisposition to the development of CAPA. These include dramatic and prolonged lymphopenia; a decrease in type I and III interferon production, which allows viral replication; excessive synthesis of tumor necrosis factor-α (TNF-α), interleukin-6 (IL-6), IL-8, and IL-10; and abnormalities in blood monocytes, such as a quantitative decrease in the membrane expression of human leukocyte antigen-D-related (mHLA-DR) on the surface of monocytes, all of which contribute to alveolar damage [1,4,18]. Additionally, some of the immunotherapies used to treat COVID-19 can increase immunosuppression and the risk of coinfections [6,19]. Thus, the administration of steroids alters phagocytosis [19,20], and the use of steroids or anti-IL6 receptors increases the risk of VAP and CAPA [8,9,21,22]. Dysregulation of the immune response has been widely described in patients with COVID-19 [1,2,23,24,25] showing in particular that severe forms of COVID-19 are associated with an increase in proinflammatory cytokines such as IL-1, IL-6 and IL-10. On the other hand, these studies highlighted that monocyte dysregulation leads to immunoparalysis shown by a decreased mHLA-DR expression. There was a correlation between poorer outcomes and high levels of proinflammatory markers/low levels of mHLA-DR, leading to the study of the IL-6/mHLA-DR ratio, which combines the two sides of immune dysregulation in COVID-19.

Immune dysregulation has been described much less in patients with CAPA [18,26]. The aim of the present study was to assess the relationship between the systemic immune response and the development of CAPA in critically ill COVID-19 patients.

## 2. Materials and Methods

### 2.1. Study Design

This was a multicenter retrospective cohort study performed in ICUs from three French hospitals in Clermont-Ferrand (one ICU), Lyon (three ICUs) and Paris (one ICU).

### 2.2. Population

From 1 March to 30 July 2020, in Paris and Lyon, and from 1 August to 31 May 2021, in Clermont-Ferrand, all adult (≥18 years) patients admitted for COVID-AHRF confirmed by RT‒PCR testing were eligible. Patients with at least one immunological assessment (following the immunological profile definition below) within the first 10 days of their ICU stay and available clinical data were included. The exclusion criteria were patients referred from another ICU and opposition from patients or their legally authorized representatives to use their medical records for medical research. Data from Paris [27] and Lyon [3] were already published with the objective of describing immunological data in severe COVID-19 patients. Having recorded the identification of *Aspergillus* infection in their database, we retrospectively added their data to the Clermont-Ferrand database in order to search for a specific link between CAPA and immunological data.

The research protocol was approved by the Ethics Committee of the French Intensive Care Society (Institutional Review Board—IRB00014135—Commission d’éthique de la société de réanimation de langue française—CE SRLF n°22-065), in accordance with French legislation, and complied with the Declaration of Helsinki and good clinical practice. Owing to its retrospective nature, no written consent was needed from the patients.

The care of COVID-19 patients was standardized across all three ICUs according to each center’s protocol, in alignment with the WHO guidelines available at the time of patient management [28].

### 2.3. Data Collection

Data, including demographic characteristics, past medical history, and biological data, were extracted from electronic case report forms at ICU admission.

Immunological profiles were generated on the basis of physician order as standard ICU practice in patients with severe COVID-19, following each center’s protocol.

The immunological profile included at least one of the following three parameters: serum IL-6, serum TNF alpha, and mHLA-DR expression. Depending on the center, the serum levels of other cytokines, such as IL-1, IL-8, and IL-10, were also measured. The techniques used in each center to measure these parameters were carried out as previously published and are given in Supplementary File S1 [1,3,27]. Owing to the use of different assay techniques, the plasma concentrations of the main interleukins (IL-6, IL-10 and TNF) were standardized by dividing the measured values by the average values obtained from healthy subjects during the validation of the assay technique used (all details are available in Supplementary File S1). Quantitative mHLA-DR expression was not normalized because the same assay technique was used at each center.

### 2.4. Aspergillus Sampling Policy

In the three centers, serum galactomannan antigen testing was performed at least once a week in all patients with COVID-19. Tracheal aspirate or nonbronchoscopic lavage with direct examination and culture were performed at least two times a week in intubated patients with COVID-19. Bronchoalveolar lavage (BAL) with galactomannan, direct examination, culture and *Aspergillus* PCR were performed by physicians at their discretion in case of suspicion of pneumonia in COVID-19-intubated patients. During bronchoscopy, 20 mL of 0.9% NaCl was inserted into single segments of the lungs four times and collected in fractions. The first, third, and fourth fractions were used for microbiological diagnostics, including cytomegalovirus (CMV) and herpes simplex virus (HSV) PCR assays. We recorded only positive results for galactomannan (in serum or BAL), direct examination or culture with *Aspergillus* or *Aspergillus* PCR (in serum or BAL).

Nonbronchoscopic lavage was performed in a blinded manner, and 10–20 mL of saline was recovered via aspiration via a closed suction system in a patient who was intubated.

### 2.5. Definitions

Possible (ps) and probable (pb) CAPA criteria were adapted from guidelines [5].

Probable CAPA requirements:

- Clinical factors (refractory fever or pleural rub or chest pain or hemoptysis or a combination).
- Compatible imagery (pulmonary infiltrate, cavitating infiltrate or both).
- Microbiology: Positive mycological direct examination of *Aspergillus* spp. on bronchoalveolar lavage (BAL) or positive mycological culture on BAL or blood galactomannan index >0.5 or BAL galactomannan index > 1.0 or two positive *Aspergillus* spp. PCRs on blood or one positive *Aspergillus* spp. PCR on BAL with Ct < 36 or an association with one positive *Aspergillus* PCR and one positive *Aspergillus* spp. PCR on BAL or a combination.

Possible CAPA requirements:

- Clinical factors (refractory fever or pleural rub or chest pain or hemoptysis or a combination).
- Compatible imagery (pulmonary infiltrate, cavitating infiltrate or both).
- Microbiology: Positive direct examination of *Aspergillus* spp. in nonbronchoscopic lavage/tracheal aspirate or positive culture of *Aspergillus* spp. on nonbronchoscopic lavage/tracheal aspirate or one galactomannan index > 4.5 on nonbronchoscopic lavage or two galactomannan index > 1.2 on nonbronchoscopic lavage or an association of one galactomannan index > 1.2 on nonbronchoscopic lavage and one positive *Aspergillus* spp. PCR in blood or nonbronchoscopic lavage or a combination.

In order to challenge actual definitions of CAPA, essentially used for research, we made our own clinical definition of “suspected CAPA” (s-CAPA):

- Compatible imagery (pulmonary infiltrate, cavitating infiltrate or both).
- Clinical factors (refractory fever or pleural rub or chest pain or hemoptysis or a combination).
- Microbiology/therapies: One positive *Aspergillus* spp. culture of sputum or one positive *Aspergillus* spp. PCR with Ct < 36 or one beta-D-glucan > 30 pg/mL and administration with anti-*Aspergillus* treatment (voriconazole, isavuconazole or liposomalamphotericin B).

Patients were classified as “CAPA”, “s-CAPA” or “non-CAPA” retrospectively on the basis of their ICU clinical files by the authors OM and MM (biological diagnosis data available in Supplementary File S1).

The immunological profiles were categorized as “D1” when the blood samples were collected within the first three ICU days and as “D7” when they were collected between the seventh and tenth days of the ICU stay. The kinetics of immunological profile parameters between D1 and D7 were defined by the D7 value of the parameter minus D1 over D1 and expressed as a percentage.

Patients were classified as having “immunosuppression” in cases of hematologic malignancy (active or in remission for less than 5 years), hematopoietic stem cell transplant for less than 5 years, active solid cancer, leukopenia < 1 G/L or neutropenia ≤ 0.5 G/L, solid organ transplantation, syndrome-acquired immunodeficiency, long-term corticosteroid therapy ≥ 0.5 mg/kg/day of prednisone equivalent for at least 3 weeks, immunosuppressive or immunomodulatory treatment.

### 2.6. Objectives of the Study

The main objective of this study was to compare the immunological profiles of the CAPA and non-CAPA groups. The second objective of the study was to study the risk factors associated with CAPA and a composite criterion including CAPA or death during the hospital stay.

### 2.7. Statistical Analysis

We performed a descriptive analysis of the data, presenting the results in the form of medians and interquartile ranges for continuous data and frequencies and percentages for categorical data. Continuous variables were dichotomized according to their median, if necessary, especially for immunological data as no real validated susceptibility threshold for CAPA already exists in the literature.

Comparisons were carried out with Wilcoxon rank-sum tests for continuous variables and chi-square tests or Fisher’s exact tests for categorical variables. A simple matching analysis with a 1:2 ratio was performed between CAPA patients and non-CAPA patients. The following criteria were used for matching: center (Clermont-Ferrand, Lyon or Paris), age (±5 years), immunosuppression status (yes/no), IMC > 30 kg/m^2^ (yes/no), corticosteroid administration during the ICU stay (yes/no), and ICU length of stay (LOS) before ps/pb CAPA events and the entire ICU stay in non-CAPA patients (ICU LOS longer than the time for the occurrence of CAPA). If multiple patients were eligible for the same pairing, those who were first admitted to the ICU were selected. Only CAPA patients successfully matched with non-CAPA patients were included in the final analysis. The standardized mean differences for the variables were reported to assess balance before and after matching.

First, simple comparisons for paired data were performed via the Wilcoxon signed-rank test for paired continuous variables, the Friedman test for repeated continuous measures, and McNemar’s test for categorical variables. Next, factors associated with CAPA occurrence were identified via univariate conditional logistic regression analyses. A multivariate conditional logistic regression analysis was performed. Variables associated with CAPA occurrence at a *p*-value < 0.10 in univariable analyses were considered as candidates for inclusion in the multivariable analysis. Given the limited number of CAPA events, an exploratory multivariable analysis was initially performed with the candidate variables. To limit model complexity and the risk of overfitting, the final conditional logistic regression model was restricted to the two covariates that remained significantly associated with CAPA occurrence (*p* < 0.05) in this analysis. This parsimonious model was used for the final multivariable analysis. The same analyses were conducted to evaluate risk factors associated with the composite outcome of CAPA or hospital death. The biological data included in the conditional logistic regression were limited to values at ICU admission (D1) or their relative changes between D1 and D7, which were calculated as follows: (D7 value − D1 value)/D1 value. Patients who developed CAPA within the first seven days of ICU admission were excluded from the analysis of interleukin kinetics between D1 and D7.

We carried out a sensitivity analysis for CAPA patients according to the FUNDICU guidelines [29] with and without matching with non-CAPA patients with a 1:3 ratio. The same criteria as described above were used for matching.

Another sensitivity analysis was carried out combining CAPA and s-CAPA patients in a “gathered CAPA” (g-CAPA) group with and without matching with non-CAPA patients with a 1:2 ratio and the same methodology.

In the absence of data concerning the exact onset of CAPA, we used a composite endpoint including CAPA and death to limit a competing risk bias (some dead patients could have developed CAPA without being diagnosed) as a secondary outcome.

For all tests, the level of significance was set at 5%. Missing data were imputed by multiple imputations with a dataset via PROC MI in SAS. Only variables with less than 30% missing data in the overall dataset were analyzed. All the statistical analyses were performed via SAS software, version 9.4 (SAS Institute, Cary, NC, USA) and R (version 3.4.0; R Core Team, Wien, Austria).

## 3. Results

### 3.1. Population

**Figure 1:**
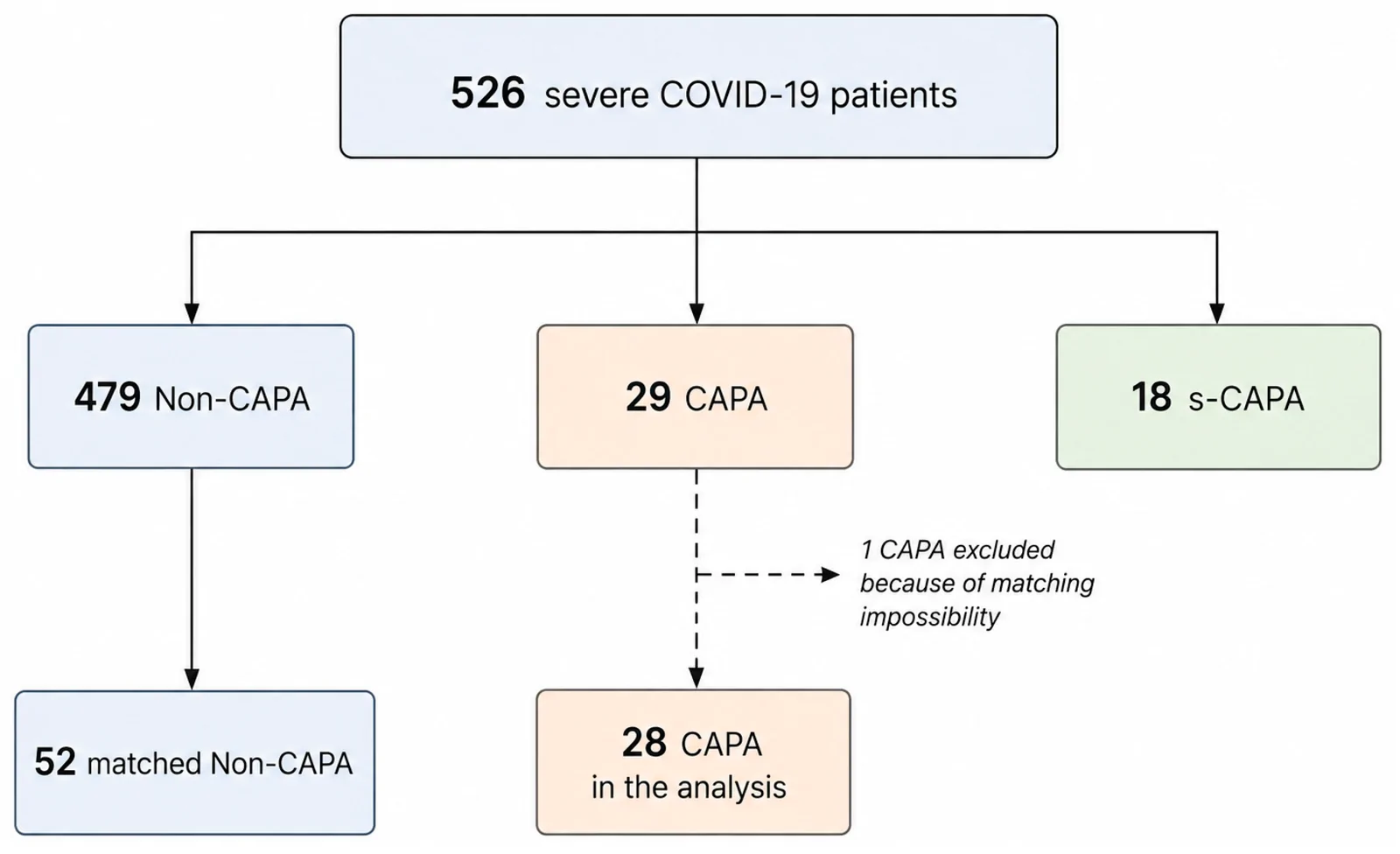
Flow chart. CAPA: COVID-19-associated pulmonary aspergillosis; ICU: intensive care unit; s-CAPA: suspected CAPA.

During the study period, 479 non-CAPA patients, 29 CAPA patients (including 22 probable CAPA and seven possible CAPA according to [5]) and 18 s-CAPA patients were included. The study flow chart is available in Figure 1. The characteristics of CAPA patients according to each center are available in Supplementary File S1.

Among the 22 probable CAPA patients, only 19 were in agreement with the FUNDICU definition of probable CAPA because of diagnosis based on PCR results. The characteristics of these patients are available in Supplementary File S1.

According to definitions, all CAPA patients were possible or probable CAPA (none proven CAPA were included in the absence of biopsy). The ICU duration of stay before the first *Aspergillus*-positive sample obtained was 8 days [7.19; 14.97] (Supplementary File S1).

The characteristics of the population are reported in Table 1. CAPA patients who required more organ support during the ICU stay (needs for vasopressors, IMV and renal replacement therapy (RRT) all *p* < 0.01) had a longer length of stay (*p* < 0.01) and higher mortality rates (*p* < 0.01).

Compared to CAPA patients, s-CAPA patients (*n* = 18) required more high-flow oxygenotherapy and less vasopressor therapy (both *p* = 0.02). There were not any other differences in clinical characteristics and laboratory results, including immunological data, mortality or length of stay. A comparison between CAPA and s-CAPA is available in Supplementary File S1.

### 3.2. Characteristics of Matched CAPA Patients and Matched Non-CAPA Patients

A comparison of the 28 CAPA patients (one CAPA patient was excluded because of matching impossibility) with the 52 matched non-CAPA patients revealed that they suffered more often from chronic respiratory disease (*p* = 0.04) on ICU admission, and during the ICU stay, they received IMV, vasopressors and RRT more frequently (all *p* < 0.01). These patients more frequently developed herpes and cytomegalovirus reactivation (both *p* < 0.01) and had a greater ICU LOS (*p* < 0.01), but there were no differences in hospital LOS or in ICU or hospital mortality (Table 2).

### 3.3. Comparison of the Immunological Profiles of Matched CAPA Patients and Matched Non-CAPA Patients

**Figure 2:**
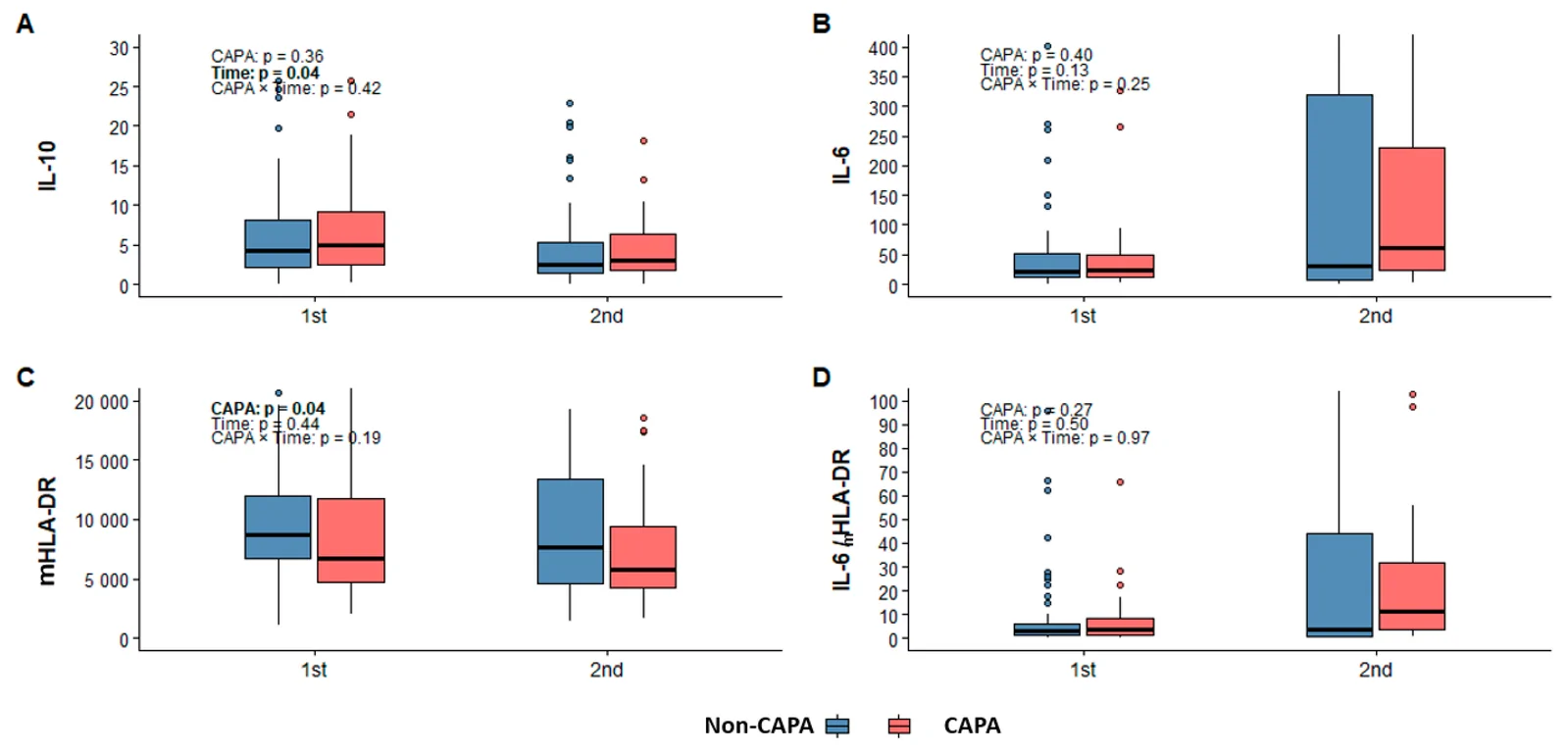
Boxplot of the inflammatory biomarkers depending on the timing of the dosage and CAPA in the cohort of 28 matched CAPA patients versus 52 matched non-CAPA patients. Boxplots representing the levels of interleukin 10 (**A**), interleukin 6 (**B**), and mHLA-DR expression (**C**) and the IL-6/mHLA-DR ratio (**D**). Interleukin levels are presented in pg/L. mHLA-DR is schematized as a count of HLA-DR on the monocyte surface;; 1st: day 1 sample; 2nd: day 7 sample; IL: interleukin; HLA: mHLA-DR. Serum levels of cytokines were previously standardized as described in the methods section.

On D1, CAPA patients had higher serum levels of IL-10 (*p* = 0.01) and IL-6 (*p* < 0.01) and a higher IL-6/mHLA-DR ratio (*p* = 0.05) than did matched non-CAPA patients. On D7, they had higher serum levels of IL-10 (*p* = 0.03) and IL-6 and a higher IL-6/mHLA-DR ratio (*p* < 0.01) but lower mHLA-DR expression (*p* < 0.01). Between D1 and D7, the kinetics (D7–D1) of the serum IL-6 concentration were greater in the matched CAPA group (*p* = 0.06), as was the IL-6/mHLA-DR ratio (*p* = 0.03) (Table 2, Figure 2).

### 3.4. Univariate Conditional Logistic Regression: Development of CAPA

The neutrophil count at ICU admission > 8 G/L (OR = 2.93, CI [1.08; 7.98], *p* = 0.04) was the only factor associated with the development of CAPA, whereas an mHLA-DR level of expression > 8000 antibodies/cell was associated with lower CAPA (OR = 0.41, CI [0.17; 0.99], *p* = 0.04) via univariate analysis. Patients with an increase in the kinetics of the IL-6/mHLA-DR ratio between D1 and D7 (OR = 3.54, CI [0.96; 13.1], *p* = 0.06), chronic respiratory failure (OR = 7.71, CI [0.88; 67.7], *p* = 0.07) or chronic kidney disease (OR = 2.99, CI [0.87; 10.19], *p* = 0.08) tended to be associated with the development of ps/pb-CAPA (Table 3). Eleven CAPA patients were not included in the analysis of interleukin kinetics between D1 and D7 because they developed CAPA during the first seven days of ICU admission.

### 3.5. Multivariate Conditional Logistic Regression

After backward selection, only two covariates remained in the final model. Chronic kidney failure (aOR 3.97, CI [1.05; 14.97], *p* = 0.04) and neutrophil count > 8 G/L (aOR 2.95, CI [1.03; 8.44], *p* = 0.04) were associated with the development of CAPA. The results of the multivariate analysis are available in Supplementary File S1. None of the immunological biomarkers remained in the final model after adjustment.

### 3.6. Sensitivity Analysis of 47 g-CAPA (CAPA and s-CAPA) Compared with 85 Matched Non-CAPA Patients with Univariate Conditional Logistic Regression

An IL-6/mHLA-DR value > 2.8 on D1 was associated with the development of CAPA (OR 3.5, CI [1.46; 8.49], *p* < 0.01), as was an increase between D1 and D7 in the IL-6/mHLA-DR ratio value (OR 3.51, CI [1.46; 8.49], *p* < 0.01). The characteristics of g-CAPA patients and their matched cohort, and the complete results of the sensitivity analysis are available in Supplementary File S1.

### 3.7. Sensitivity Analysis of 19 Probable CAPA Patients According to FUNDICU Definition Compared with 56 Matched Non-CAPA Patients with Univariate Conditional Logistic Regression

None of the patients’ characteristics, ICU therapies nor immunological biomarkers were associated with the development of CAPA. The characteristics of probable CAPA according to FUNDICU and their matched cohort, and the results of the sensitivity analysis are available in Supplementary File S1.

#### Composite Criteria: Development of CAPA or Death

The presence of chronic respiratory failure (OR 9.1, IC [1.06; 78.44], *p* = 0.04) and a serum concentration of IL-10 > 3.2 pg/L on D1 (OR 4.08, CI [1.09; 15.38], *p* = 0.04) was associated with the development of CAPA or death. An IL-6/mHLA-DR ratio > 2.8 on D1 (OR 3.16, IC [0.98; 10.21], *p* = 0.05) and a serum concentration of IL-6 > 15.8 pg/L on D1 (OR 3.49, IC [0.94; 13.05], *p* = 0.06) tended to be associated with the development of CAPA or death. mHLA-DR expression > 8000 antibodies/cell on D1 was associated with lower development of CAPA or death (OR 0.26, IC [0.08; 0.81], *p* = 0.02) (Table 4).

### 3.8. Sensitivity Analysis of 47 g-CAPA (CAPA and s-CAPA) Patients Compared with 85 Matched Non-CAPA Patients with Univariate Conditional Logistic Regression

An increase in the IL-6/mHLA-DR ratio value between D1 and D7 was associated with the development of CAPA or death (OR 3.85, CI [1.48; 10.04], *p* = 0.01) whereas an increase in the mHLA-DR value between D1 and D7 tended to be associated with lower CAPA or death (OR 0.12, CI [0.02; 0.96], *p* = 0.05). The results of the sensibility analysis are available in Supplementary File S1.

## 4. Discussion

In our study, we observed that the development of CAPA was associated with dysregulation of the equilibrium between immune activation and suppression in patients with severe COVID-19 admitted to the ICU. First, the serum levels of IL-10 and IL-6, and the IL-6/mHLA-DR ratio observed on both D1 and D7 were greater in the CAPA group, whereas mHLA-DR expression was lower. In the univariate analysis, while a value of mHLA-DR expression > 8000 at ICU admission was associated with lower development of CAPA, an IL-6/mHLA-DR ratio > 2.8 at admission (*p* = 0.05), a serum concentration of IL-10 > 3.2 pg/L (*p* = 0.04) and an increase in the IL-6/mHLA-DR ratio between D1 and D7 were almost associated with developing CAPA or death. None of the immunological parameters were associated with CAPA in the multivariate analysis.

In the literature, the rates of CAPA in ICU patients range between 2.5% and 47.2% [6,8,9,10,11], which is consistent with our findings, which revealed an incidence of 5.5% for CAPA patients (8,9% including s-CAPA). However, discrepancies between the studies concerning patient characteristics, rates of invasive mechanical ventilation, treatments administered for COVID-19, virus strains, screening policies for respiratory fungal microorganisms, and CAPA definitions make comparisons difficult.

In our study, CAPA patients developed more organ failure, more often required organ support during their ICU stay, and had longer hospital stays and increased mortality. The development of invasive pulmonary aspergillosis (IPA) is a well-known risk factor for mortality in immunocompromised patients, regardless of whether they are hospitalized in the ICU or have hematological malignancies or a viral lung disease such as influenza [11,20,30,31,32,33,34]. The MYCOVID study [6] reported similar findings in a cohort of 565 COVID-19 patients on mechanical ventilation, of whom 76 (15%) had proven or probable CAPA. In this study, patients with proven or probable CAPA had significantly higher SOFA scores, longer durations of mechanical ventilation and increased mortality. Similarly, in a recent study of 335 patients with severe COVID-19 requiring mechanical ventilation, Feys et al. reported a proven or probable CAPA incidence of 33% (significantly higher in the vaccination era) and a higher 90-day mortality in the CAPA group [10].

The IPA and CAPA are entities that are difficult to define accurately, as shown by the numerous definitions used in the literature [20,29,31,34,35,36]. Recently, Basseti et al. published the FUNDICU guidelines defining proven and probable IPA in the ICU [29]. In these guidelines, the chosen definition of probable IPA is based notably on the results of bronchoalveolar lavage without including PCR results to improve the comparability and reproducibility of research studies. CAPA mortality rates differ according to the definitions used [37]. Sometimes, there is no difference in mortality rates between different categories within a single classification; for example, mortality in patients with proven aspergillosis is no greater than that in patients with suspected aspergillosis, which raises the question of the relevance of the classification [30]. In patients with COVID-19, the survival benefit of antifungal treatment in patients with CAPA remains unclear [6].

In our study, there was no case of proven CAPA because lung biopsy cultures were not performed in the ICUs participating in the study during the COVID-19 pandemic. We tried to challenge all these definitions, essentially created to promote research, with a more clinical practice one, whereby our “s-CAPA” included all patients who were treated with anti-*Aspergillus* therapy because of clinical and radiological suspicion associated with a non-conventional mycological sample (such as positive direct examination/culture of non-BAL respiratory sample or positive Beta-D-Glucan). There were no significant differences in the outcomes nor the immunological results between CAPA and s-CAPA patients in our study, in regard to this small population of 19 s-CAPA patients. Moreover, univariate analysis of g-CAPA patients and their matched non-CAPA population highlighted once more an association between the increase in mHLA-DR ratio and the development of g-CAPA, as suggested by the analysis of only CAPA patients. However, our study is still underpowered to really allow the questioning of the actual guideline definition.

The classical risk factors for IPA in critically ill patients include hematological malignancies, organ transplantation, systemic corticosteroid administration and, to a lesser extent, underlying respiratory diseases, cardiovascular disease, diabetes mellitus and viral pneumonia [11,31]. In our study, chronic respiratory disease tended to be a CAPA risk factor, which is in agreement with previously published results on CAPA [11,33]. Most of the patients in both the CAPA and non-CAPA groups received corticosteroids as recommended [38]. Corticosteroids are known to predispose patients to IPA [11,29,33]. To avoid bias, we matched patients according to immunosuppression status and corticosteroid administration, leaving both the CAPA group and the matched non-CAPA group with >90% receiving corticosteroids during the ICU stay. In the MYCOVID study, the risk factors for developing CAPA were age > 62 years, a combination of dexamethasone and anti-interleukin 6 therapies, and duration of mechanical ventilation > 14 days [6]. More recently, Feys et al. reported ICU admission during the vaccination era, age, corticosteroid use during the ICU stay, and EORTC/MSGERC host factor(s) as CAPA risk factors via binary logistic regression [10]. Host factors such as moderate/severe COPD, decompensated cirrhosis, uncontrolled HIV infection and solid tumors were also described in the FUNDICU guidelines [29].

Severe cases of COVID-19 are associated with a significant increase in the serum IL-6 concentration and the IL-6/mHLA-DR ratio, indicating a dysregulated balance between immune activation and suppression, which leads to severe pulmonary inflammation and acute respiratory distress syndrome [1]. Concerning the development of invasive pulmonary aspergillosis (IPA), Feys et al. described a three-level breach in antifungal immunity in influenza associated with pulmonary aspergillosis and CAPA that affects the integrity of the epithelial barrier, the capacity to phagocytize and kill *Aspergillus* spores, and the ability to destroy *Aspergillus* hyphae, which is mainly mediated by neutrophils [18]. In our study, IL-6 serum levels at D1 and D7 were greater in CAPA patients than in non-CAPA patients. Similarly, the mHLA-DR counts were lower at D7 in both the CAPA and ps/pb CAPA groups than in the non-CAPA matched population, leading to a higher IL-6/mHLA-DR ratio in these groups. In univariate analysis, a higher expression of mHLA-DR at ICU admission was associated with lower development of CAPA, whereas an increase in the kinetics of the IL-6/mHLA-DR ratio between admission and D7 was almost associated with the development of CAPA. Taken together, these findings are in agreement with the results of Camargo et al. suggesting the predominant role of inflammation driven by IL-6 [17] and its consequences in terms of immune suppression expressed by low mHLA-DR values, as reported in previous works [1]. HLA-DR is the main antigen-presenting molecule for helper T cells on the surface of monocytes, macrophages and dendritic cells. Thus, the decrease in mHLA-DR on the surface of blood monocytes may reflect a reduced overall capacity for presenting antigens and therefore for inducing adaptative immune responses. Low mHLA-DR has become one of the best markers of monocyte immunosuppressive phenotypes in various diseases, such as cancer and sepsis [39,40]. Consequently, the use of the IL-6/mHLA-DR ratio could be of interest for simultaneously analyzing the two aspects of the immune response in the same patient, as suggested by Bonnet et al. [1].

In univariate analysis, a serum concentration of IL-10 at admission > 3.2 pg/L was associated with the development of CAPA or death. This increase in IL-10 appears to be multifactorial, partially because of a feedback loop initiated by the increased IL-6 levels observed in patients with severe COVID-19 [1] and potentially partly driven by *Aspergillus* infection itself [41].

IL-6 has the capacity to reduce the expression of mHLA-DR on the surface of monocytes [42,43]. IL-10 likewise facilitates the internalization of HLA-DR molecules within monocytes, especially in septic shock [44]. This downregulation compromises the effective presentation of antigens to the immune system, impairing the host’s ability to initiate an appropriate immune response against invading pathogens. In addition, owing to its anti-inflammatory properties, IL-10 exerts suppressive effects on T lymphocyte activation, proinflammatory cytokine production, and macrophage function [14].

Together, the concurrent downregulation of mHLA-DR and increased IL-10 levels may contribute to immune exhaustion in these patients, thereby increasing susceptibility to *Aspergillus*. Several murine studies have shown increased susceptibility to IPA in the presence of elevated IL-10 levels [12,14]. Conversely, resistance to the disease has been observed in cases of IL-10 deficiency [13]. Although observational, our results are consistent with the current understanding of *Aspergillus* infection.

Our research is novel in terms of serum immune profiling data in patients with severe COVID-19, and our cohort of over 526 patients stands apart from other studies in the current literature, although results from Paris and Lyon were already published without focusing on CAPA. Another strength of our study lies in the systematic matching of CAPA patients, which we performed to limit potential confusion bias in our analyses.

However, several limitations need to be acknowledged. First, only 29 patients with CAPA were identified (and only 28 matched for analysis), likely reflecting the inclusion of patients from Paris and Lyon during the first wave of the COVID-19 pandemic in France, whereas the main center contributed patients from later waves. In addition, the use of invasive mechanical ventilation decreased over time due to evolving management strategies and improved outcomes, despite mechanical ventilation duration being a well-established risk factor for CAPA [6]. Consequently, a type II error cannot be excluded given the limited sample size, as reflected by wide confidence intervals and borderline *p* values.

Furthermore, the absence of the FUNDICU consensus criteria at the time of data collection may have led to misclassification bias. We retrospectively conducted a sensitivity analysis using these criteria but with the few probable CAPA patients according to FUNDICU in our study (*n* = 19) we were not able to find a link between immunological profile and the development of CAPA. During the study period, each center applied its own mycological sampling strategy, introducing heterogeneity in diagnostic practices and a potential underdiagnosis of CAPA depending on the frequency and type of respiratory sampling. Moreover, bronchoalveolar lavage (BAL) data were not systematically recorded in the non-CAPA group, limiting the ability to fully exclude undiagnosed CAPA in controls and potentially contributing to misclassification bias, which may partly explain the relatively low observed CAPA incidence. The difference in invasive mechanical ventilation requirements between CAPA and non-CAPA patients (89.7% vs. 34.8%) is probably due to limited access to BAL and so contributed to potential CAPA underdiagnosis.

Although matching by center mitigated the impact of inter-center variability and wave-related effects in diagnostic and therapeutic strategies, residual confounding may persist, particularly regarding within-center temporal changes in clinical practice. Indeed, patients from later waves were less frequently intubated and more often received immunomodulatory therapies such as dexamethasone and tocilizumab, which may have influenced both susceptibility to secondary infections and immunological profiles [45,46]. Additionally, differences in cytokine assay platforms across centers may have introduced measurement variability; however, the use of standardized cytokine values (IL-6, IL-10, TNF-α) partially mitigates this limitation and allows for inter-center comparisons.

Third, missing data limit the robustness of the findings and warrant validation in larger, preferably prospective, multicenter cohorts, particularly including a higher number of patients with proven or probable aspergillosis. The absence of precise timing of CAPA diagnosis is another important limitation, as some patients may have met diagnostic criteria earlier during their ICU stay than recorded. To address this issue, a simple logistic regression model was used. In addition, a composite endpoint including CAPA and death was introduced to limit a competing risk bias, as some deceased patients could have developed CAPA without being diagnosed, although a competitive risk model such as Fine Gray would have been more appropriate (but impossible due to missing data concerning CAPA onset).

A potential confounding factor in the interpretation of immunological data is the occurrence of secondary infections during the ICU stay, which are frequent in severe COVID-19 [47,48] and may themselves be promoted by both viral-induced immune dysregulation and immunomodulatory treatments. However, the lack of precise timing of infectious complications limits causal inference regarding their relationship with immune profiles and CAPA onset. Similarly, the absence of data on the timing of organ dysfunction restricts the ability to disentangle whether immunological alterations are associated with, or secondary to, disease severity. Although matching on duration of ICU stay prior to CAPA diagnosis helped reduce immortal time and exposure bias, residual confounding cannot be excluded. In the absence of a previous validated threshold concerning CAPA predisposal, we used medians to dichotomize patients for immunological data, which could have inflated type 1 error.

Importantly, reverse causality remains a major limitation: the observed differences in immune profiles at day 7 may reflect the consequences of evolving CAPA and clinical deterioration rather than predisposing immunological signatures. The retrospective design further limits causal inference and the generalizability of the findings.

## 5. Conclusions

This study did not identify an association between the immunological profile and the development of CAPA in multivariate analysis. However, the link between mHLA-DR expression, the serum concentration of IL-10, the IL-6/mHLA-DR ratio and the development of CAPA may be interesting directions for future studies with prospective larger cohorts, as suggested by univariate analysis.

## Figures and Tables

**Table 1:** Characteristics of the entire population. The results are presented as the median [CI] or value (%). ^a^: years; ^b^: mmHg; ^c^: days. BMI: body mass index; CV: cardiovascular; ECMO: extracorporeal membrane oxygenation; HFNC: high-flow nasal cannula; ICU: intensive care unit; IMV: invasive mechanical ventilation; LOS: length of stay; N: number; RRT: renal replacement therapy; SAPS II: simplified acute physiology score. Immunosuppression was defined as the following: hematologic malignancy (active or in remission for less than 5 years), hematopoietic stem cell transplant for less than 5 years, active solid cancer, leukopenia < 1 G/L or neutropenia ≤ 0.5 G/L, solid organ transplantation, syndrome-acquired immunodeficiency, long-term corticosteroid therapy ≥ 0.5 mg/kg/day of prednisone equivalent for at least 3 weeks, and immunosuppressive or immunomodulatory treatment.

	Non-CAPA		CAPA		*p* Value
	N = 479	% missing	N = 29	%missing	
Time from symptoms to admission ^c^	9 [6; 12]	4	9 [6; 11]	0	0.93
Time from hospital to ICU admission ^c^	2 [1; 4]	0	1 [1; 5]	0	0.62
Age ^a^	66.2 [56.57; 73.79]	0	72 [66; 76]	0	<0.01
Gender (male)	334 (69.73)	0	20 (69.0)	0	0.93
BMI > 30 kg/m^2^	180 (37.6)	0	13 (44.8)	0	0.43
Main comorbidities		0		0	
Chronic CV disease	76 (15.9)	0	7 (24.1)	0	0.25
Chronic respiratory disease	47 (9.9)	0	5 (17.2)	0	0.20
Chronic renal disease	53 (11.1)	0	9 (31.0)	0	<0.01
Immunosuppression	80 (16.7)	0	7 (24.1)	0	0.30
Diabetes	91 (19.1)	0	6 (20.7)	0	0.83
Immunomodulatory treatment	14 (4.3)	32	2 (8.3)	17	0.37
Characteristics during D1–D2				0	
SAPS II	36 [29; 45]	13	43 [34; 52]	0	0.02
PaO2/FiO2 ^b^	100 [72.22; 150]	12	80 [61; 122]	0	0.10
IMV	115 (24.0)	0	13 (44.9)	0	0.01
Non-IMV	35 (7.4)	0	1 (3.5)	0	0.43
HFNC	229 (48.3)	1	14 (48.3)	0	1.00
RRT	17 (3.7)	2	4 (13.8)	0	<0.01
Vasopressors	105 (22.9)	4	12 (44.4)	6	0.01
Corticosteroids	285 (59.5)	0	23 (79.3)	0	0.03
During ICU stay				0	
Vasopressor	159 (33.5)	0	27 (93.1)	0	<0.01
IMV	166 (34.8)	0	26 (89.7)	0	<0.01
ECMO	6 (1.5)	14	1 (3.9)	10	0.35
RRT	66 (13.9)	0	18 (62.1)	0	<0.01
Corticosteroids	314 (74.6)	12	25 (89.3)	3	0.08
Tocilizumab	11 (2.3)	0	4 (13.79)	0	0.20
ICU LOS ^c^	7 [4; 11]	0	19 [13; 29]	0	<0.01
ICU death	106 (22.1)	0	17 (58.6)	0	<0.01
Hospital LOS ^c^	13 [9; 22]	8	20 [15; 55]	0	<0.01
Hospital death	174 (36.3)	0	20 (69.0)	0	<0.01

**Table 2:** Comparison of matched CAPA patients and matched non-CAPA patients. The results are presented as the median [CI] or value (%). ^a^: years; ^b^: mmHg; ^c^: days. ^d^: G/L; ^e^: ng/mL; ^f^: pg/L; ^g^: antibodies/cell; ^h^: (day 7 value minus day 1 value) over day 1 value. BMI: body mass index; CV: cardiovascular; D1: sample during the first ICU, three days; D7: sample from the ICU, seven to 10 days; ECMO: extracorporeal membrane oxygenation; HFNC: high-flow nasal cannula; HLA: human leukocyte antigen;; ICU: intensive care unit; IL: interleukin; IMV: invasive mechanical ventilation; LOS: length of stay; N: number; RRT: renal replacement therapy; SAPS II: simplified acute physiology score;; TNF: tumor necrosis factor. Serum levels of cytokines were previously standardized as described in the methods section on immunosuppression: hematologic malignancy (active or in remission for less than 5 years), hematopoietic stem cell transplant for less than 5 years, active solid cancer, leukopenia < 1 G/L or neutropenia ≤ 0.5 G/L, solid organ transplantation, syndrome-acquired immunodeficiency, long-term corticosteroid therapy ≥ 0.5 mg/kg/day of prednisone equivalent for at least 3 weeks, and immunosuppressive or immunomodulatory treatment.

	Matched Non-CAPA		Matched CAPA		*p* Value
	N = 52	% missing	N = 28	% missing	
Time from symptoms to admission	9 [5; 13]	0	9 [6; 11]	0	0.55
Time from hospital to ICU admission	2 [1; 5]	0	1 [1; 5]	0	0.63
Age ^a^	71 [65.7; 78.1]	0	72 [66; 76]	0	0.81
Gender (male)	36 (69.2)	0	20 (69)	0	0.98
BMI > 30 kg/m^2^	21 (40.4)	0	13 (44.8)	0	0.70
Main comorbidities				0	
Chronic CV disease	10 (19.2)	0	7 (24.1)	0	0.60
Chronic respiratory disease	2 (3.8)	0	5 (17.2)	0	0.04
Chronic renal disease	7 (13.5)	0	9 (31)	0	0.06
Immunosuppression	8 (15.4)	0	7 (24.1)	0	0.33
Diabetes	9 (17.3)	0	6 (20.7)	0	0.71
Characteristics on D1–D2					
SAPS II	41 [33; 50]	1	43 [34; 52]	0	0.75
PaO2/FiO2 ^b^	84.1 [72.4; 117.2]	0	80 [61; 122]	0	0.50
IMV	22 (42.3)	0	13 (44.8)	0	0.83
Non IMV	3 (5.8)	0	1 (3.4)	0	0.64
HFNC	24 (46.2)	0	14 (48.3)	0	0.85
RRT	4 (8)	3	4 (13.8)	0	0.41
Vasopressors	16 (32)	3	12 (44.4)	7	0.28
Corticosteroids	43 (82.7)	0	23 (79.3)	0	0.71
During ICU stay					
Vasopressors	27 (51.9)	0	27 (93.1)	0	<0.01
IMV	29 (55.8)	0	26 (89.7)	0	<0.01
ECMO	0 (0)	9	1 (3.8)	10	0.18
RRT	12 (23.1)	0	18 (62.1)	0	<0.01
Corticosteroids	46 (95.8)	7	25 (89.3)	0	0.27
Tocilizumab	3 (5.8)	0	4 (13.8)	0	0.22
ICU LOS ^c^	10 [8; 15.5]	0	19 [13; 29]	0	<0.01
ICU Death	23 (44.2)	0	18 (62.1)	0	0.12
Hospital LOS	17 [13; 24]	1	20 [15; 55]	0	0.07
Hospital death	27 (51.9)	0	20 (69)	0	0.14
Laboratory features on D1					
Neutrophils ^d^	7162.5 [4315; 10,717]	7	9820 [6825; 14,050]	32	0.12
Lymphocytes ^d^	600 [500; 950]	7	650 [300; 900]	32	0.52
Neutrophils/lymphocytes	16.7 [9.1; 30.4]	7	12.8 [10; 26.8]	32	0.86
Platelets ^d^	203 [162; 267]	1	235 [154; 288]	28	0.42
D-dimers ^e^	1200 [1000; 2800]	11	1300 [800; 4100]	35	0.82
IL-10 ^f^	3 [1.5; 5.9]	9	6.1 [3.5; 9.4]	7	<0.01
IL-6 ^f^	15 [6; 31.6]	3	24.6 [14.1; 52.9]	0	0.02
TNF-α ^f^	1 [0; 2]	5	0.2 [0; 2.4]	3	0.55
mHLA-DR ^g^	9392 [7347; 11,647]	26	6633.4 [5586; 10,819]	25	0.15
IL-6/mHLA-DR	1.4 [0.5; 3.7]	28	3.2 [2.3; 4.5]	25	<0.01
Laboratory features on D7					
IL-10 ^f^	2.1 [1.1; 4.1]	23	3.2 [1.9; 7]	17	0.03
IL-6 ^f^	14 [5.7; 40]	17	48.1 [22; 122.3]	14	<0.01
TNF-α ^f^	0.6 [0; 1.8]	23	0.6 [0; 1.4]	17	0.78
mHLA-DR ^g^	7658.6 [4988; 11,837]	30	5166 [3719; 5959]	28	<0.01
IL-6/mHLA-DR	1.7 [0.5; 7.9]	30	9 [3.1; 24.5]	28	<0.01
ΔIL-10 ^h^	−0.3 [−0.7; 0.1]	26	−0.2 [−0.7; 0]	17	0.92
ΔIL-6 ^h^	0 [−0.6; 1.8]	17	0.3 [−0.1; 3]	14	0.06
ΔmHLA-DR ^h^	0 [−0.4; 0.7]	30	−0.4 [−0.6; 0]	28	0.09
ΔIL-6/mHLA-DR ^h^	0 [−0.8; 2.5]	30	1.2 [0.2; 5.2]	28	0.03

**Table 3:** Risk factors for CAPA in the matched CAPA + matched non-CAPA population—univariate conditional logistic regression. ^a^: Value on D1; ^b^: (day 7 value minus day 1 value) over day 1 value. BMI: body mass index; CI: confidence interval; CKD: chronic kidney disease; CV: cardiovascular; HLA: human leukocyte antigen; IL: interleukin; RRT: renal replacement therapy; PCT: procalcitonin. The serum levels of cytokines were standardized as described in the methods section.

Variable	OR	OR CI	*p* Value
Gender (male)	1.08	[0.42; 2.78]	0.87
BMI > 30 kg/m^2^	1.17	[0.46; 3.00]	0.74
Chronic CV disease	1.37	[0.46; 4.05]	0.58
Chronic respiratory failure	7.71	[0.88; 67.7]	0.07
CKD	2.99	[0.87; 10.19]	0.08
Diabetes	1.17	[0.40; 3.44]	0.78
RRT	1.69	[0.42; 6.85]	0.46
Vasopressors	4.90	[0.57; 42.41]	0.14
Neutrophils > 8 G/L ^a^	2.93	[1.08; 7.98]	0.04
Lymphocytes > 0.6 G/L ^a^	1.45	[0.60; 3.54]	0.41
Neutrophils/lymphocytes > 10 ^a^	1.52	[0.56; 4.11]	0.41
D-dimers > 1500 ng/mL ^a^	0.95	[0.33; 2.78]	0.93
PCT > 0.5 µg/L ^a^	1.27	[0.40; 4.08]	0.69
IL-10 > 3.2 pg/L ^a^	2.27	[0.84; 6.16]	0.11
IL-1 > 0.4 pg/L ^a^	0.55	[0.21; 1.47]	0.23
IL-6 > 15.8 pg/L ^a^	2.00	[0.73; 5.49]	0.17
TNF- α > 1 pg/L ^a^	0.66	[0.22; 2.03]	0.47
mHLA-DR > 8000 antibodies/cell ^a^	0.41	[0.17; 0.99]	0.042
IL-6/mHLA-DR > 2.8 ^a^	1.94	[0.83; 4.55]	0.13
ΔIL-6 > 0 ^b^	1.69	[0.58; 4.90]	0.35
ΔmHLA-DR > 0 ^b^	0.22	[0.05; 1.10]	0.07
ΔIL-6/mHLA-DR > 0 ^b^	3.54	[0.96; 13.1]	0.06

**Table 4:** Risk factors for CAPA or death in the matched CAPA + matched non-CAPA population—univariate conditional logistic regression. ^a^: D1 value; ^b^: (day 7 value minus day 1 value) over day 1 value. BMI: body mass index; CKD: chronic kidney disease; CV: cardiovascular; HLA: human leukocyte antigen; IL: interleukin; IMV: invasive mechanical ventilation; RRT: renal replacement therapy; PCT: procalcitonin. The serum levels of cytokines were standardized as described in the methods section.

Variable	OR	OR IC	*p* Value
Gender (male)	2	[0.64; 6.24]	0.23
BMI > 30 kg/m^2^	0.78	[0.27; 2.26]	0.65
Chronic CV disease	1.21	[0.28; 5.2]	0.8
Chronic respiratory failure	9.1	[1.06; 78.44]	0.04
CKD	3.94	[0.74; 20.89]	0.11
Diabetes	1.1	[0.26; 4.71]	0.9
IMV	1.1	[0.26; 4.71]	0.9
RRT	1.69	[0.23; 12.23]	0.61
Remdesivir	1.36	[0.2; 9]	0.75
Tocilizumab	4	[0.36; 44.11]	0.26
Neutrophils > 8 G/L ^a^	2.66	[0.85; 8.27]	0.09
Lymphocytes > 0.6 G/L ^a^	0.84	[0.27; 2.63]	0.77
Neutrophils/lymphocytes > 10 ^a^	1.98	[0.62; 6.32]	0.25
D-dimers > 1500 ng/mL ^a^	3.1	[0.63; 15.38]	0.17
PCT > 0.5 µg/L ^a^	3.14	[0.34; 28.58]	0.31
IL-10 > 3.2 pg/L ^a^	4.08	[1.09; 15.36]	0.04
IL-1 > 0.4 pg/L ^a^	0.49	[0.12; 2.02]	0.32
IL-6 > 15.8 pg/L ^a^	3.49	[0.94; 13.05]	0.06
TNF- α > 1 pg/L ^a^	1.94	[0.52; 7.25]	0.32
mHLA-DR > 8000 antibodies/cell ^a^	0.26	[0.08; 0.81]	0.02
IL-6/mHLA-DR > 2.8 ^a^	3.16	[0.98; 10.21]	0.05
ΔIL-10 > 0 ^b^	0.57	[0.18; 1.82]	0.34
ΔIL-6 > 0 ^b^	2.03	[0.58; 7.14]	0.27
ΔmHLA-DR > 0 ^b^	3.71	[0.97; 14.27]	0.06

## Data Availability

The datasets used and/or analyzed during the current study are available from the corresponding author upon reasonable request.

## Supplementary Materials

The following supporting information can be downloaded at https://www.mdpi.com/article/10.3390/jof12100763/s1. Supplementary File S1: Immune profile and risk of COVID-19-associated pulmonary aspergillosis (CAPA): the CYTOCAPA study.

## Abbreviations

The following abbreviations are used in this manuscript:

BMI	Body mass index
CAPA	COVID-19-associated pulmonary aspergillosis
CKD	Chronic kidney disease
COVID-19	SARS-CoV-2-associated disease
CV	Cardiovascular
D1	Period from day 1 to day 3 after ICU admission
D7	Period from day 7 to day 10 after ICU admission
ECMO	Extracorporeal membrane oxygenation
HFNC	High-flow nasal cannula
ICU	Intensive care unit
IL	Interleukin
IMV	Invasive mechanical ventilation
IPA	Invasive pulmonary aspergillosis
LOS	Length of stay
mHLA-DR	Human leukocyte antigen-DR expression on monocytes
no-CAPA	Severe COVID-19 population who did not develop CAPA
PCT	Procalcitonin
ps/pb CAPA	Possible or probable CAPA
SAPS	Simplified acute physiology score
SOFA	Sequential organ failure assessment
