# Supplementary material for: Immune Profile and Risk of COVID-19-Associated Pulmonary Aspergillosis (CAPA): A Multicenter Retrospective Study with Matching Cohorts
Source: J Fungi (Basel). 2026 Oct 10;12(10):763. doi: 10.3390/jof12100763 (PMC13706347; doi:10.3390/jof12100763)
Supplement: Supplementary file 1 [file jof-12-00763-s001.zip › jof-4426850-File S1.pdf]

Immune profile and risk of Covid-19 associated pulmonary aspergillosis (CAPA): the CYTOCAPA study

Supplemental file

Summary

1. **Immune profile assessment methodology** **2**
2. **Ethics** **4**
3. **Definitions of CAPA** **4**
4. **Full methods** **4**
5. **Full study data (including number of missing data)** **9**
   a. Description of the main paper population 9
   b. Description of the whole cohort 10
   c. Laboratory results of the whole cohort 12
   d. Analysis of CAPA versus matched cohort M1no-CAPA 13
   e. Analysis of CAPA and matched cohort M1no-CAPA: laboratory results 14
   f. Risk factors of CAPA in the population CAPA and M1no-CAPA 15
   g. Risk factors of CAPA or death in the population CAPA and M1no-CAPA 16
   h. Subgroup analysis of ps/pb-CAPA and no-CAPA populations 17
   i. Subgroup analysis of ps/pb-CAPA and no-CAPA: laboratory results 19
   j. Subgroup analysis of ps/pb-CAPA and s-CAPA populations 20
   k. Subgroup analysis of ps/pb-CAPA and s-CAPA: laboratory results 21
   l. Analysis of ps/pb-CAPA and matched no-CAPA (M2no-CAPA) populations 22
   m. Analysis of ps/pb-CAPA and matched no-CAPA (M2no-CAPA): laboratory results 23
   n. Risk factor of ps/pb CAPA in the ps/pb CAPA + matched cohort M2no-CAPA population– conditional logistic regression 24
   o. Risk factor of ps/pb CAPA in the ps/pb CAPA + matched cohort M2no-CAPA population– conditional logistic regression (multivariate analysis) 24
   p. Risk factor of ps/pb CAPA or death in the ps/pb CAPA + matched cohort M2no-CAPA population– conditional logistic regression (multivariate analysis) 25
   q. Flow chart of the main paper 26
   r. Flow chart of the whole population 27
   s. Boxplot of the inflammatory biomarkers depending on the timing of the dosage and CAPA in the cohort of 47 CAPA versus x matched no-CAPA cases (M1no-CAPA) 28

t. Boxplot of the inflammatory biomarkers depending on the timing of the dosage and CAPA in the cohort of 29 ps/pb CAPA versus 52 matched cases (M2no-CAPA) 29
u. Biological diagnosis data...30
v. Description of the main population according by center...30
w. Description of CAPA patients according to FUNDICU guidelines and matched Non-CAPA patients 32
x. Risk factor of FUNDICU CAPA in the FUNDICU CAPA and matched cohort – Univariate conditionnal logictic regression 35

1. Immune profile assessment methodology

a. Main centre (Clermont-Ferrand)

Analysis of HLA-DR expression on monocytes

Monocyte HLA-DR (mHLA-DR) expression, in numbers of antibodies bound per monocyte (Ab/cell), was analysed. EDTA-blood was drawn at D1 and D7 after inclusion of patients in the study. The blood was stored at 4-8°C and processed within 4 h after withdrawal. The quantitative expression of mHLA-DR was determined with the Anti-HLA-DR/Anti-Monocyte QuantiBRITE assay (BD Biosciences, San Jose, CA, USA). Briefly, whole blood (25 µL) was stained with 10 µL of QuantiBRITE HLADR/Monocyte mixture (QuantiBRITE antiHLADR PE (clone L243)/Antimonocytes (CD14) PerCPCy5.5 (clone MfP9), Becton Dickinson San Jose, CA, USA) at room temperature for 30 min in a dark chamber. Samples were then lysed using FACS Lysing Solution (Becton Dickinson San Jose, CA, USA) for 15 min. After a washing step, cells were acquired on a BD LSR II cytometer (BD Biosciences, San Jose, CA, USA) and flow data were analysed with FlowLogic software (version 7.3 software, Miltenyi Biotec, Germany). The total number of antibodies bound per monocyte (Ab/cell), defined as total CD14+ cells, were quantified by calibration with a standard curve determined with BD QuantiBRITE phycoerythrin (PE) beads (BD Biosciences, San Jose, CA, USA).

Plasma cytokine analysis

Plasma was obtained by centrifugation of EDTA samples within 4 h of phlebotomy. Plasma was placed in aliquots and stored at 20°C until use. Human pro-inflammatory cytometric bead array (CBA) and human IFN-a flex kits (BD Biosciences, San Jose, CA, USA) were used. The human pro-inflammatory cytokine kit simultaneously detects IL-6, IL-10 and CXCL8 cytokines in a single sample and the Interferon-a flex kit detects Interferon-a. The assays were performed according to the manufacturer's instructions. Samples and standards were acquired on the BD LSR II cytometer (BD Biosciences, San Jose, CA, USA) and the generated FSC files were analysed with FCAP Array version 3.0 software. Plasma IL-1Ra levels were measured with a commercial enzyme-linked immunoassay (ELISA) kit (human IL-1Ra ELISA from Invitrogen, Villebon-sur-Yvette, France). Plasma samples were frozen and stored for batch analysis. Briefly, to determine IL-1Ra levels, samples were thawed and 50 µL aliquots were incubated in microtitre wells coated with anti-human IL-1Ra antibody. The wells were then washed and detection achieved by adding biotin-conjugated antihuman IL-1Ra antibody followed by incubation with streptavidinHRP, and finally by addition of horseradish peroxidase (HRP) substrate solution. A coloured product formed in proportion to the amount of human IL-1Ra present and absorbance was measured at 450 nm.

b. Paris centre

Serum IL-6, IL-1b, tumor necrosis factor alpha (TNFα), and IL-8 were analyzed according to the manufacturer's instructions (Ella, ProteinSimple).

Monocyte HLA-DR (mHLA-DR) expression, in numbers of antibodies bound per monocyte (Ab/cell), was analysed. EDTA-blood was drawn at D1 and D7 after inclusion of patients in the study. The blood was stored at 4-8°C and processed within 4 h after withdrawal. The quantitative expression of mHLA-DR was determined with the Anti-HLA-DR/Anti-Monocyte QuantiBRITE assay (BD Biosciences, San Jose, CA, USA). Briefly, whole blood (25 µL) was stained with 10 mL of QuantiBRITE HLADR/Monocyte mixture (QuantiBRITE antiHLADR PE (clone L243)/Antimonocytes (CD14) PerCPCy5.5 (clone MfP9), Becton Dickinson San Jose, CA, USA) at room temperature for 30 min in a dark chamber. Samples were then lysed using the FACS Lysing solution (Becton Dickinson San Jose, CA, USA) for 15 min. After a washing step, cells were acquired on a BD LSR II cytometer (BD Biosciences, San Jose, CA, USA) and flow data were analysed with FlowLogic software (version 7.3 software, Miltenyi Biotec, Germany). The total number of antibodies bound per monocyte (Ab/cell), defined as total CD14+ cells, were quantified by calibration with a standard curve determined with BD QuantiBRITE phycoerythrin (PE) beads (BD Biosciences, San Jose, CA, USA).

c. Lyon centre

Blood samples

Ethylene diamine tetra acetic acid (EDTA-)anticoagulated blood was drawn five times during the first month after ICU admission: within the first 48 h after admission (Day 0: D0); between 72 and 96 h after admission (D3); between D7 and D9 (D7); between D12 and D15 (D12); and between D20 and D25 (D20). Blood was stored at 4–8 °C and processed within 4 h after withdrawal.

Cytokine measurement

Whole blood was sampled in EDTA tubes and plasma was frozen at − 20 °C within 4 h following blood collection. Cytokines were measured in batches after one freeze/thaw cycle using standardized protocols meeting the accreditation requirements for clinical and diagnostic laboratories of the International Organization for Standardization. Plasma concentrations of IL-6, TNF-α, IFN-γ and IL-10 were measured by Simpleplex® technology using ELLA instrument (ProteinSimple®, San Jose, CA) following the manufacturer's instructions. Plasma IFNα2 concentrations were determined by single-molecule Array (SIMOA®) on a HD-1 Analyzer (Quanterix) using a commercial kit for IFN-α2 quantification (Quanterix, Lexington, Mass).

Flow cytometry

T lymphocyte subpopulation immunophenotyping was performed on an automated volumetric flow cytometer from Beckman Coulter (Aquios CL) as previously described [19]. Monocyte HLA-DR expression and B and NK immunophenotyping were performed using antibodies from Beckman-Coulter and BD Biosciences. The expression of monocyte HLA-DR was determined using the Anti-HLA-DR/Anti-Monocyte Quantibrite assay (BD Biosciences, San Jose, USA). The total number of antibodies bound per cell (AB/C) was quantified using calibration with a standard curve determined with BD Quantibrite phycoerythrin (PE) beads (BD Biosciences) as described elsewhere [20]. B and NK lymphocyte immunophenotyping was performed using lyophilized antibody panel from Beckman Coulter (Duraclone kit). Data were acquired on a Navios flow cytometer (Beckman Coulter, Hialeah, FL), and flow data were analyzed using Navios software (Beckman Coulter). Enumeration of lymphocyte subpopulations and mHLA-DR measurement were performed using standardized protocols meeting the accreditation requirements for clinical and diagnostic laboratories of the International Organization for Standardization.

d. Standardization of laboratory values

Due to different assay techniques, plasmatic concentrations of main interleukins (IL-6, IL-10 and TNFa) were standardized by dividing the measured values by the normal threshold values obtained from healthy subjects during the validation of the assay technique used.

Main centre (Clermont-Ferrand) used Human pro-inflammatory cytometric bead array (CBA) to quantify serum concentration of interleukins. Normal interleukin serum concentrations validated with this technique in healthy subjects are:

IL-6 < 2.4 pg/mL

IL-10 < 1.4 pg/mL

TNF alpha < 1.1 pg/mL

(Data from the Laboratoire d'Immunologie – CHU Clermont-Ferrand).

Serum concentrations of IL-6, IL-10 and TNFa from Clermont-Ferrand centre were divided by these thresholds.

Lyon and Paris centres used the kit Ella Protein Simple to quantify serum concentration of interleukins. Normal interleukin serum concentrations validated with this technique in healthy subjects are :

IL-6 < 7 pg/mL

IL-10 < 8 pg/mL

TNF alpha < 10 pg/mL

(Data from the Laboratoire d'Immunologie – Hospices Civils de Lyon).

Serum concentrations of IL-6, IL-10 and TNFa from Clermont-Ferrand centre were divided by these thresholds.

mHLA-DR expression was quantified using the same technique in each centre so we didn't need to standardize the results.

We did not find any normal values of IL-1 recorded in healthy subjects with the techniques used so we did not report the serum concentration of IL-1 measured in the main manuscript.

2. Ethics

The research protocol was approved by the Ethics Committee of the French Intensive Care Society (FICS; CE SRLF n°22-065).

Data from Lyon were already published (Venet F, Cour M, Rimmelé T, et al.: Longitudinal assessment of IFN-I activity and immune profile in critically ill COVID-19 patients with acute respiratory distress syndrome. *Crit Care Lond Engl* 2021; 25:140) with a research protocol approved by Comité de Protection des Personnes Ile de France 1 - N°IRB/IORG #: IORG0009918) under agreement number 2020-A01079-30.

Data from Paris were already published (Dupont T, Caillat-Zucman S, Fremeaux-Bacchi V, et al.: Identification of Distinct Immunophenotypes in Critically Ill Coronavirus Disease 2019 Patients. *Chest* 2021; 159:1884–1893) with a research protocol approved by Ethics Committee of the French Intensive Care Society (FICS; CE SRLF n°20-32).

3. Definitions of CAPA

Definitions of probable and possible CAPA were taken from the guidelines of Koehler P, Bassetti M, Chakrabarti A, Chen SCA, Colombo AL, Hoenigl M, et al. Defining and managing COVID-19-associated pulmonary aspergillosis: the 2020 ECMM/ISHAM consensus criteria for research and clinical guidance. Lancet Infect Dis. juin 2021;21(6):e149-62.

Probable CAPA requirements:

- Compatible imagery (pulmonary infiltrate, cavitating infiltrate ou both)
- Clinical factors (refractory fever or pleural rub or chest pain or haemoptysis or a combination)
- Microbiology: positive mycological direct exam of *aspergillus* spp on BAL or positive mycological culture on BAL or blood galactomannan index >0.5 or BAL galactomannan index > 1.0 or two positive *aspergillus* spp PCR on blood or one positive *aspergillus* spp PCR on BAL with Ct < 36 or an association with one positive *aspergillus* PCR and one positive *aspergillus* spp PCR on BAL or a combination

Possible CAPA requirements:

- Compatible imagery (pulmonary infiltrate, cavitating infiltrate ou both)
- Clinical factors (refractory fever or pleural rub or chest pain or haemoptysis or a combination)
- Microbiology: positive direct examination of *aspergillus* spp in non bronchoscopic lavage or positive *aspergillus* spp culture on non guided bronchoscopic lavage or one galactomannan index > 4.5 on non guided bronchoscopic lavage or two galactomannan index > 4.5 on non guided bronchoscopic lavage or an association of one galactomannan index > 1.2 on non guided bronchoscopic lavage and one positive *aspergillus* spp PCR in blood or non guided bronchoscopic lavage or a combination

Suspected CAPA requirements (created for the study):

- Compatible imagery (pulmonary infiltrate, cavitating infiltrate ou both)
- Clinical factors (refractory fever or pleural rub or chest pain or haemoptysis or a combination)
- Microbiology/therapies: One positive *aspergillus* spp culture of sputum or one positive *aspergillus* spp PCR with Ct <36 or one beta-D-glucan > 30 pg/mL and administration with anti-*aspergillus* treatment (voriconazole, isavuconazole or liposomalamphotericin B)

4. **Full methods**

Study design

This was a multicentre retrospective cohort study performed in ICUs from three French Hospitals in Clermont-Ferrand (one ICU), Lyon (three ICUs) and Paris (one ICU).

Population

From March 1 to July 30, 2020 in Paris and Lyon, and from August 1 to May 31, 2021 in Clermont-Ferrand, all adult (≥18 years) patients admitted for COVID-AHRF confirmed by RT-PCR testing were eligible. Patients with an immunological profile assessment performed at least once within the first 10 days of ICU stay were included. Exclusion criteria were patients referred from another ICU and opposition from patients or their legally authorized representatives to use their medical records for medical research. The research protocol was approved by the Ethics Committee of the French Intensive Care Society (FICS; CE SRLF n°22-065).

Data collection

Data were extracted from electronic case report forms on ICU admission including demographic characteristics, past medical history, biological data,

The immunological profile included at least one the following three parameters: serum IL-6, serum TNF alpha, and mHLA-DR expression. Depending on the centres, serum levels of other cytokines such as IL-1, IL-8, and IL-10 were also measured. The techniques used in each centre to measure these parameters were carried out as previously published and are given in Supplemental file (1, 3, 24). Due to different assay techniques, plasmatic concentrations of main interleukins (IL-6, IL-10 and TNF) were standardized by dividing the measured values by the average values obtained from healthy subjects during the validation of the assay

technique used (all details are available in Supplemental file). Quantitative mHLA-DR expression was not normalized due to the use of the same assay technique in each center.

Definitions

Possible (ps) and probable (pb) CAPA were defined according to guidelines (5) (Supplemental file). Suspected CAPA (s-CAPA) was arbitrarily defined by identification of *Aspergillus spp* by a positive culture of sputum yielding *Aspergillus spp* or by PCR in respiratory sample with Ct > 36. Patients with a positive beta-D-glucan (>30 pg/mL) and administered with anti-*Aspergillus spp* treatment i.e. voriconazole, isavuconazole or liposomal amphotericin B with no other cause for receiving the treatment than suspected CAPA were also included in the s-CAPA group. Finally, the g-CAPA group (gathered CAPA) was defined by the combination of both s-CAPA and ps/pb-CAPA subgroups. Patients were classified "s-CAPA", "ps/pb-CAPA" or "non-CAPA" retrospectively on the basis of their ICU clinical file by OM and MM (biological diagnosis data available in Supplemental file).

Immunological profiles were categorized as "D1", when samples were processed within the first three ICU days, and as "D7", when processed between the seventh and the tenth day of ICU stay. The kinetics of immunological profile parameters between D1 and D7 was defined by the D7 value of the parameter minus D1 over D1, and expressed as a percentage.

Objective of the study

The main objective of the study was to compare the immunological profile of CAPA and non-CAPA groups. The secondary objective was to compare the immunological profiles of ps/pb-CAPA and non-CAPA patients.

Statistical analysis

We performed a descriptive analysis of the data, presenting the results in the form of median and interquartile range for continuous data and frequency and percentage for categorical data. Comparisons were carried out

with Wilcoxon rank-sum tests for continuous variables, and Chi 2 tests or Fisher's exact tests for categorical variables. A simple matching analysis with a 1:2 ratio was performed between CAPA and non-CAPA (M1no-CAPA) patients. A subgroup analysis was performed of ps/pb-CAPA and non-CAPA patients (M2no-CAPA), including a matching analysis with a 1:2 ratio between CAPA and non-CAPA (M2no-CAPA) patients. The following criteria were used for both matchings: centre (Clermont-Ferrand, Lyon or Paris), age (+/- 5 years), immunosuppression status (yes/no), IMC > 30 kg/m² (yes/no), corticosteroid administration during the ICU stay (yes/no), and ICU length of stay (LOS) before ps/pb or suspected CAPA events and the entire ICU stay in non-CAPA patients (ICU LOS longer than time for occurrence of CAPA). First, simple comparisons for paired data were made using Wilcoxon signed-rank test for paired continuous data, Friedman test for continuous data, and McNemar's test for categorical data. Then, factors associated with CAPA occurrences were then sought for using univariate conditional logistic regression analyses with imputation of missing data. A multivariate conditional logistic regression analysis was performed, including varia-bles with a p-value <0.10 in the univariate analyses. Same analysis were made to evaluate risk factors associated with the composite criterion CAPA or hospital death. We used medians of interleukin concentrations as threshold values during the conditional logistic regression. Data of patients who developed CAPA within the first seven days of ICU admission were not considered from the risk factors analysis of interleukin concentration kinetics between D1 and D7.

We made a sensitivity analysis for CAPA patients according to the FUNDICU guidelines with and without matching with non-CAPA patients with a 1:3 ratio. The same criteria described higher where used for matching.

Another sensitivity analysis was made combining CAPA and s-CAPA patients in a "gathered CAPA" (g-CAPA) group with and without matching with non-CAPA patients with a 1:2 ratio and the same methodology.

For all tests, the level of significance was set at 5%. Missing data were imputed by multiple imputations with a dataset via PROC MI in SAS. Only variables with less than 30% miss-ing data were analyzed. All the statistical analyses were performed via SAS software, ver-sion 9.4 (SAS Institute, Cary, NC) and R (version 3.4.0; R Core Team, Wien, Austria).

5. **Full study data (including number of missing data)**

a. Description of the main paper population

	No CAPA (n=479)	n of missing data	CAPA (n=29)	n of missing data	p-value
**Time from symptoms to admission**	9 [6; 12]	22	9 [6; 11]	0	0.93
**Time from hospital to ICU admission**	2 [1; 4]	0	1 [1; 5]	0	0.62
**Age[a]**	66.2 [56.57; 73.79]	0	72 [66; 76]	0	<0.01
**Gender (male)**	334 (69.729)	0	20 (68.966)	0	0.93
**BMI > 30 kg/m²**	180 (37.578)	0	13 (44.828)	0	0.43
Main comorbidities					
**Chronic CV disease**	76 (15.933)	2	7 (24.138)	0	0.25
**Chronic respiratory disease**	47 (9.853)	2	5 (17.241)	0	0.20
**Chronic renal disease**	53 (11.065)	0	9 (31.034)	0	<0.01
**Immunosuppression**	80 (16.701)	0	7 (24.138)	0	0.30
**Diabetes**	91 (19.078)	2	6 (20.69)	0	0.83
**Immunomodulatory treatment**	14 (4.334)	156	2 (8.333)	5	0.37
Characteristics during D1-D2					
**SAPS II**	36 [29; 45]	65	43 [34; 52]	0	0.02
**SOFA**	5 [4; 6]	156	5 [5; 6.5]	0	0.06
**Worst PaO2/FiO2[b]**	100 [72.22; 150]	58	80 [61; 122]	0	0.10
**IMV**	115 (24.008)	0	13 (44.828)	0	0.01
**Non-IMV**	35 (7.368)	4	1 (3.448)	0	0.43
**HFNC**	229 (48.312)	5	14 (48.276)	0	1.00
**RRT**	17 (3.656)	14	4 (13.793)	0	<0.01
**Vasopressors**	105 (22.876)	20	12 (44.444)	2	0.01
**Corticosteroids**	285 (59.499)	0	23 (79.31)	0	0.03
During ICU stay					
**Vasopressor**	159 (33.474)	4	27 (93.103)	0	<0.01
**IMV**	166 (34.801)	2	26 (89.655)	0	<0.01
**ECMO**	6 (1.463)	69	1 (3.846)	3	0.35
**RRT**	66 (13.866)	3	18 (62.069)	0	<0.01
**Corticosteroids**	314 (74.584)	58	25 (89.286)	1	0.08
**Tocilizumab**	11 (2.296)	0	4 (13.793)	0	0.20
**ICU LOS[d]**	7 [4 ; 11]	0	19 [13 ; 29]	0	<0.01
**ICU Death**	106 (22.129)	0	17 (58.621)	0	<0.01
**Hospital LOS[d]**	13 [9 ; 22]	42	20 [15 ; 55]	0	<0.01
**Hospital death**	174 (36.326)	0	20 (68.966)	0	<0.01

Results are presented as median [IC] or value (%). a: years; b: mmHg, c: positive PCR on ≥1 blood sample; d: days. BMI: body mass index, CMV: cytomegalovirus, CV: cardiovascular, ECMO extracorporeal membrane oxygenation, HFNC: high-flow nasal cannula, HSV: herpes simplex virus, ICU: Intensive Care Unit, IMV: invasive mechanical ventilation, LOS: length of stay, n: number, RRT: renal replacement therapy; SAPS II: simplified acute physiology score, SOFA: sequential organ failure assessment. Immunosuppression: Hematologic malignancy (active or in remission for less than 5 years), hematopoietic stem cell transplant for less than 5 years, active solid cancer, leukopenia < 1 G/L or neutropenia ≤ 0.5 G/L, solid organ transplantation, syndrome acquired immunodeficiency, long-term corticosteroid therapy ≥ 0.5 mg/kg/day of prednisone equivalent for at least 3 weeks, immunosuppressive or immunomodulatory treatment

b. Description of the whole cohort

	Non-CAPA (n=479)	g-CAPA (n=47)	p-value
**Time from symptoms to admission**	9 [6;12]<br>*Miss = 22*	9 [6;11]	0.56
**Time from hospital to ICU admission**	2 [1;4]	2 [;5]	0.28
**Age**[a]	66.2 [56.6;73.8]	70.3 [62;75.2]	0.02
**Gender (male)**	334 (69.7)	35 (74.5)	0.50
**BMI > 30 kg/m²**	180 (37.6)	22 (46.8)	0.21
Main comorbidities			
**Chronic CV disease**	76 (15.9)<br>*Miss = 2*	13 (27.7)	0.04
**Chronic respiratory disease**	47 (9.9)<br>*Miss = 2*	8 (17)	0.13
**Chronic renal disease**	53 (11.1)	11 (23.4)	0.01
**Immunosuppression**	80 (16.7)	10 (21.3)	0.43
**Diabetes**	91 (19.1)<br>*Miss = 2*	10 (21.3)	0.72
**Immunomodulatory treatment**	14 (4.3)<br>*Miss = 156*	3 (6.3)<br>*Miss = 5*	0.42
Characteristics during D1-D2			
**SAPS II**	36 [29;45]<br>*Miss = 65*	41 [29;48]	0.09
**SOFA**	5 [4;6]<br>*Miss = 156*	5 [5;7]	0.02
**Worst PaO2/FiO2**[b]	100 [72.2;150]	77 [60;118.3]	<.01
**IMV**	115 (24)	15 (31.9)	0.23
**Non-IMV**	35 (7.4)<br>*Miss = 4*	3 (6.4)	0.8
**HFNC**	229 (48.3)<br>*Miss = 5*	29 (61.7)	0.08
**RRT**	17 (3.7)<br>*Miss = 14*	4 (8.5)	0.11
**Vasopressors**	105 (22.9)<br>*Miss = 20*	14 (31.1)	0.21
**Corticosteroids**	284 (59.5)	36 (76.6)	0.02
During ICU stay			
**Vasopressor**	159 (33.5)<br>*Miss = 4*	43 (91.5)	<.01
**IMV**	166 (34.8)<br>*Miss = 2*	42 (89.4)	<.01
**ECMO**	6 (1.5)<br>*Miss = 69*	2 (4.5)<br>*Miss = 3*	0.14
**RRT**	66 (13.9)<br>*Miss = 3*	25 (53.2)	<.01
**Corticosteroids**	314 (74.6)<br>*Miss = 58*	42 (91.3)<br>*Miss = 1*	<.01
**Tocilizumab**	34 (7.3)<br>*Miss = 12*	6 (12.8)	0.18
**ICU LOS**[d]	7 [4;11]	21 [14;38]	<.01
**ICU Death**	106 (22.1)	29 (61.7)	<.01
**Hospital LOS**[d]	13 [9;22]<br>*Miss = 42*	27 [17;56]	<.01
**Hospital death**	174 (36.3)	32 (68.1)	<.01

Results are presented as median [IC] or value (%). a: years; b: mmHg, c: positive PCR on ≥1 blood sample, d: G/L, e: ng/mL, f: pg/L, g: mHLA-DR count on monocyte, h: (Day 7 value minus Day 1 Value) of Day 1 value, i: days. BMI: Body mass index, CMV: Cytomegalovirus, CV: cardiovascular, ECMO Extracorporeal membrane oxygenation, HFNC: High flow nasal cannula, HLA: Human leukocyte antigen, HSV: Herpes simplex virus, ICU: Intensive care unit, IL: Interleukin, IMV: Invasive mechanical ventilation, LOS: Length of stay, RRT: Renal replacement therapy; SAPS II: Simplified acute physiology score, SOFA: Sequential organ failure assessment, TNF: Tumor necrosis factor. Immunosupression: Hematologic malignancy (active or in remission for less than 5 years), hematopoietic stem cell transplant for less than 5 years, active solid cancer, leukopenia < 1 G/L or neutropenia ≤ 0.5 G/L, solid organ transplantation, syndrome acquired immunodeficiency, long-term corticosteroid therapy ≥ 0.5 mg/kg/day of prednisone equivalent for at least 3 weeks, immunosuppressive or immunomodulatory treatment.

c. Laboratory results of the whole cohort

	Non CAPA (n=479)	g-CAPA (n=47)	p-value
Laboratory features on ICU admission	Median [IC]	Median [IC]	
**Neutrophils**[a]	7000 [5000;9960]<br>*Miss = 82*	9200 [6900;12000]<br>*Miss = 10*	<.01
**Lymphocytes**[a]	795 [500;1100]<br>*Miss = 83*	600 [400;900]<br>*Miss = 10*	<.01
**Neutrophils/lymphocytes**	9.3 [5.7; 14.4]<br>Miss=83	13.3 [9.2; 28.6]<br>Miss=10	<.01
**Platelets**[a]	223 [169.5;295]<br>*Miss = 35*	222.5 [154;288]<br>*Miss = 9*	0.79
**D-dimers**[b]	1290 [800;2500]<br>*Miss = 82*	1300 [850;2633]<br>*Miss = 11*	0.75
**IL-10**[c]	3 [1.5;5.9]<br>*Miss = 111*	4.6 [2.4;8.6]<br>*Miss = 3*	<.01
**IL-6** [c]	15 [6;31.6]<br>*Miss = 26*	23.4 [11.8;50.5]<br>Miss = 1	<.01
**TNF-α** [c]	1 [0;2]<br>*Miss = 35*	0.2 [0;1.9]<br>*Miss = 2*	0.29
**mHLA-DR**[d]	10182 [7003;14354]<br>*Miss = 188*	6645 [4962;12032]<br>*Miss = 10*	<.01
**Il6 /mHLA-DR**	1.4 [0.5;3.7]<br>*Miss = 198*	3.1 [1.7;6.5]<br>*Miss = 10*	<0.01
Laboratory features after one week			
**IL-10** [c]	2.1 [1.1;4.1]<br>*Miss = 259*	8.5 [4.3;17.3]<br>*Miss = 8*	0.02
**IL-6** [c]	14 [5.7;40]<br>*Miss = 190*	45.7 [15.2;105.8]<br>*Miss = 7*	<0.01
**TNF- α** [c]	0.6 [0;1.8]<br>*Miss = 263*	0.5 [0;1.4]<br>*Miss = 8*	0.98
**mHLA-DR**[d]	8130.5 [5052;13958.5]<br>*Miss = 291*	5129 [3612.7;6182]<br>*Miss = 15*	<.01
**Il6 /mHLA-DR**	1.7 [0.5;7.9]<br>*Miss = 292*	8.4 [2.7;24.4]<br>*Miss = 15*	<0.01
**ΔIL-10**[e]	-0.3 [-0.7;0.1]<br>*Miss = 265*	-0.3 [-0.6;0]<br>*Miss = 8*	0.88
**ΔIL-6** [e]	0 [-0.6;1.8]<br>*Miss = 194*	0.4 [-0.4;3.1]<br>*Miss = 7*	0.05
**ΔmHLA DR** [e]	-0.1 [-0.5;0.2]<br>*Miss = 291*	-0.3 [-0.6;0]<br>*Miss = 15*	0.09
**ΔDelta IL-6/mHLA DR** [e]	-0.1 [-0.8;2.3]<br>*Miss = 292*	0.7 [0;3.2]<br>*Miss = 15*	0.06

a: G/L, b: ng/mL, c: pg/L, d: mHLA-DR count on monocyte,, e: (Day 7 value minus Day 1 value) of Day 1 value. HLA: human leukocyte antigen, IL: interleukin, TNF: tumor necrosis factor. Serum levels of cytokines were previously standardized as described in the method section.

d. Analysis of CAPA versus matched cohort M1no-CAPA

	M1no-CAPA (N=85)	g-CAPA (N = 47)	P value
**Time from symptoms to admission**	9 [5;12]<br>*Miss = 6*	9 [6;11]	0.95
**Time from hospital to ICU admission**	2 [1 ; 4]	2 [1 ; 5]	0.52
**Age[a]**	69.6 [64.5;75.4]	70.3 [62;75.4]	0.73
**Gender (Male)**	60 (70.6)	35 (74.5)	0.63
**BMI > 30 kg/m²**	36 (42.4)	22 (46.8)	0.62
**Main comorbidities**			
**Chronic CV disease**	15 (17.6)	13 (27.7)	0.18
**Chronic respiratory disease**	4 (4.7)	8 (17)	0.02
**Chronic renal disease**	10 (11.8)	11 (23.4)	0.08
**Immunosuppression**	10 (11.8)	10 (21.3)	0.14
**Diabetes**	13 (15.3)	10 (21.3)	0.39
Characteristic during D1-D2			
**SAPS II**	38 [32;47]<br>*Miss = 1*	41 [29;48]	1.00
**SOFA**	6 [5;10]<br>*Miss =52*	5 [5;7]	0.34
**PaO2/FiO2[b]**	80 [63;106.2]	77 [60;118.3]	0.71
**IMV**	25 (29.4)	15 (31.9)	0.76
**Non IMV**	6 (7.1)	3 (6.4)	0.88
**HFNC**	48 (56.5)	29 (61.76)	0.56
**RRT**	5 (6)<br>*Miss = 2*	4 (8.5)	0.59
**Vasopressors**	20 (24.1)<br>*Miss = 2*	14 (31.1)<br>*Miss = 2*	0.39
**Corticosteroids**	68 (80)	36 (76.6)	0.65
During ICU stay			
**Vasopressors**	44 (51.8)	43 (91.5)	<0.01
**IMV**	45 (52.9)	42 (89.4)	<0.01
**ECMO**	0 (0)<br>*Miss = 5*	2 (4.5)<br>*Miss = 3*	0.05
**RRT**	19 (22.4)	25 (53.2)	<0.01
**Corticosteroids**	72 (97.3)<br>*Miss = 11*	42 (91.3)<br>*Miss = 1*	0.14
**Tocilizumab**	6 (7.1)	6 (12.8)	0.27
**HSV[c]**	4 (4.7)	16 (34)	<0.01
**CMV[c]**	1 (1.2)	8 (17)	<0.01
**ICU LOS[d]**	10 [8;16]	21 [14;38]	<0.01
**ICU Death**	35 (41.2)	30 (63.8)	0.01
**Hospital LOS**	17 [13;26]<br>*Miss = 1*	27 [17;56]	<0.01
**Hospital Death**	40 (47.1)	32 (68.1)	0.02

Results are presented as median [IC] or value (%). a: years; b: mmHg, c: positive PCR on ≥1 blood sample; d: days. BMI: Body mass index, CMV: Cytomegalovirus, CV: cardiovascular, ECMO Extracorporeal membrane oxygenation, HFNC: High flow nasal cannula, HSV: Herpes simplex virus, ICU: Intensive care unit, IMV: Invasive mechanical ventilation, LOS: Length of stay, RRT: Renal replacement therapy; SAPS II: Simplified acute physiology score, SOFA: Sequential organ failure assessment

e. Analysis of CAPA and matched cohort M1no-CAPA : laboratory results

	Matched cohort (N=85)	g-CAPA (N = 47)	pvalue
**Laboratory features on ICU admission**			
**Neutrophils**[a]	8000 [5100 ; 11000] *Miss = 7*	9200 [6900 ; 12000] *Miss = 10*	0.15
**Lymphocytes**[a]	700 [500 ; 1050] Miss = 7	600 [400 ; 900] Miss = 10	0.07
**Neutrophils/lymphocytes**	11.2 [7.1 ; 18.3] Miss=7	13.3 [9.2 ; 28.6] Miss=10	0.04
**Platelets**[a]	206.5 [177 ; 264] Miss = 3	222.5 [154 ; 288] Miss = 9	0.51
**D-dimers**[b]	1200 [900 ; 2200] Miss = 8	1300 [850 ; 2633.3] Miss = 11	0.82
**IL-10**	3.2 [1.7 ; 6.8] Miss = 6	4.6 [2.4 ; 8.6] Miss = 3	0.05
**IL-6**	15.3 [6.9 ; 28.9] Miss = 3	23.4 [11.8 ; 50.5] Miss = 1	0.01
**TNF-α**	1.7 [0.9 ; 2.5] Miss = 4	0.2 [0 ; 1.9] Miss = 2	<0.01
**mHLA DR**[d]	9166 [6595 ; 121997] Miss = 26	66459 [4962 ; 12032] Miss = 10	0.15
**IL-6/mHLA DR**	2.3 [0.9 ; 4.1] Miss=27	3.1 [1.7 ; 6.5] Miss=10	0.06
**Laboratory features after one week**			
**IL-10**	2 [1.1 ; 3.8] Miss = 18	3 [1.9 ; 5.7] Miss = 8	0.04
**IL6**	13 [5.9 ; 30.6] Miss = 15	45.7 [15.2 ; 105.8] Miss = 7	<0.01
**TNF-α**	1 [0 ; 2.4] Miss = 18	0.5 [0 ; 1.4]Miss = 8	0.18
**mHLA DR**[d]	7760 [4723.5 ; 13389.5] Miss = 25	5129 [3612.7 ; 6182] Miss = 15	<0.01
**Il6/mHLA DR**	1.7 [0.6 ; 7.9]	8.4 [2.7 ; 24.4]	<0.01
**ΔIL-10**[e]	-0.4 [-0.6 ; 0.2] Miss = 25	-0.3 [-0.6 ; 0] Miss = 15	0.95
**ΔIL-6**[e]	-0.1 [-0.6 ; 1.2] Miss = 15	0.4 [-0.4 ; 3.1] Miss = 7	0.03
**ΔmHLA DR**[e]	-0.1 [-0.5 ; 0.4] Miss = 25	-0.3 [-0.6 ; 0] Miss = 15	0.13
**ΔIL-6/mHLA DR**[e]	0 [-0.6 ; 2.8] Miss = 25	0.9 [0 ; 4] Miss = 15	0.39

a: G/L, b: ng/mL, c: pg/L, d: mHLA-DR count on monocyte, e: (Day 7 value minus Day 1 Value) of Day 1 value. HLA: Human leukocyte antigen, Il: Interleukin, TNF: Tumor necrosis factor. Serum levels of cytokines were previously standardized as described in the method section.

f. Risk factors of CAPA in the population CAPA and M1no-CAPA – conditional logistic regression

Subgroup	OR	IC	P value
**Age > 70 years**	2.00	[0.46 ; 8.62]	0.35
**Gender (male)**	1.249	[0.55 ; 2.81]	0.59
**BMI > 30 kg/m²**	1.15	[0.55 ; 2.39]	0.71
**Chronic liver Failure**	0.78	[0.07 ; 8.88]	0.84
**Chronic CV Disease**	1.55	[0.66; 3.63]	0.31
**Chronic respiratory failure**	3.53	[0.86 ; 14.1]	0.07
**CKD**	1.95	[0.71 ; 5.36]	0.19
**Diabetes**	1.16	[0.47 ; 2.87]	0.75
**RRT**	1.27	[0.33 ; 4.80]	0.72
**Vasopressors**	2.52	[0.61 ; 10.27]	0.20
**Tocilizumab**	4.33	[0.82 ; 22.82]	0.08
**Neutrophils > 8 G/L**[a]	1.38	[0.68 ; 2.80]	0.37
**Lymphocytes > 0.6 G/L**[a]	0.94	[0.46 ; 1.9]	0.86
**Neutrophils/Lymphocytes >10**	1.26	[0.61; 2.62]	0.54
**D-dimers > 1500 ng/mL**[a]	0.97	[0.45 ; 2.13]	0.95
**PCT >0.5 µg/L**[a]	1.24	[0.54 ; 2.88]	0.61
**IL-10 > 3.2** [a]	1.30	[0.60 ; 2.86]	0.51
**IL-6 > 15.8**[a]	1.13	[0.49 ; 2.58]	0.78
**TNF-α > 1.2 pg/L**[a]	0.66	[0.29 ; 1.49]	0.32
**mHLA DR > 8000** [a]	0.63	[0.31 ; 1.24]	0.18
**IL-6/mHLA DR >2.8** [a]	3.51	[1.46; 8.49]	<0.01
**ΔIL-6 > 0** [b]	1.66	[0.76 ; 3.64]	0.20
**ΔIL-6/mHLA DR** [b]	3.51	[1.46 ; 8.49]	<0.01

a: Value on D1, b: (Day 7 value minus Day 1 Value) of Day 1 value. BMI: Body mass index, CKD: Chronic kidney disease, CV: Cardiovascular, HLA: Human leukocyte antigen, IL: Interleukin, IMV: Invasive mechanical ventilation, mHLA-DR: HLA-DR count on monocyte, RRT: Renal replacement therapy, PCT: Procalcitonin. Serum levels of cytokines were previously standardized as described in the method section.

g. Risk factors of CAPA or death in the population CAPA and M1no-CAPA – conditional logistic regression

Variable	OR	IC	p-value
**Gender (male)**	2	[0.77; 5.17]	0.15
**BMI > 30 kg/m²**	0.85	[0.35; 2.03]	0.71
**Chronic CV Disease**	1.31	[0.47; 3.63]	0.61
**Chronic respiratory failure**	2.9	[0.7; 12.06]	0.14
**CKD**	3.96	[1.01; 15.58]	0.05
**Diabetes**	0.82	[0.23; 2.85]	0.75
**RRT**	1.69	[0.23; 12.23]	0.61
**Remdesivir**	0.3	0.06; 1.56]	0.15
**Tocilizumab**	2.73	[0.47; 15.91]	0.26
**Neutrophils > 8 G/L**[a]	1.36	[0.61; 3.02]	0.46
**Lymphocytes > 0.6 G/L**[a]	0.62	[0.26; 1.47]	0.27
**Neutrophils/Lymphocytes >10**[a]	1.86	[0.75; 4.63]	0.18
**D-dimers > 1500 ng/mL**[a]	1.24	[0.5; 3.08]	0.65
**PCT >0.5 µg/L**[a]	1.94	[0.63; 5.93]	0.25
**IL-10 > 6.8 pg/L**[a]	2.29	[0.89; 5.9]	0.09
**IL-1 > 0.4 pg/L**[a]	0.79	[0.27; 2.27]	0.66
**IL-6 > 50 pg/L**[a]	1.85	[0.67; 5.06]	0.23
**TNF-α > 1.2 pg/L**[a]	1.13	[0.43; 2.91]	0.81
**mHLA-DR > 8000**[a]	0.59	[0.27; 1.33]	0.2
**IL6/mHLA-DR > 5.8**[a]	1.52	[0.66; 3.51]	0.33
**ΔIL-10> 0**[b]	0.52	[0.19; 1.43]	0.21
**ΔIL-6 > 0** [b]	2.58	[0.96; 6.94]	0.06
**ΔIL-6/mHLA-DR > 0** [b]	3.85	[1.48; 10.04]	0.01
**ΔmHLA-DR > 0** [b]	0.12	[0.02; 0.96]	0.05

a: Value on D1, b: (Day 7 value minus Day 1 Value) of Day 1 value. BMI: Body mass index, CKD: Chronic kidney disease, CV: Cardiovascular, HLA: Human leukocyte antigen, IL: Interleukin, IMV: Invasive mechanical ventilation, mHLA-DR: HLA-DR count on monocyte, RRT: Renal replacement therapy, PCT: Procalcitonin. Serum levels of cytokines were previously standardized as described in the method section.

h. Subgroup analysis of ps/pb-CAPA and non-CAPA populations

	Non-CAPA (n = 479)	Ps/pb-CAPA (n=29)	P value
**Time from symptoms to admission**	9 [6;12] *Miss = 22*	9 [6;11]	0.93
**Time from hospital to ICU admission**	2 [1;4]	1 [1;5]	0.62
**Age[a]**	66.2 [56.6 ; 73.8]	72 [66 ; 76]	<0.01
**Gender (Male)**	334 (69.7)	20 (69)	0.93
**BMI > 30 kg/m²**	180 (37.6)	13 (44.8)	0.43
Main comorbidities			
**Chronic CV disease**	76 (15.9) *Miss = 2*	7 (24.1)	0.25
**Chronic respiratory disease**	47 (9.9) *Miss = 2*	5 (17.2)	0.20
**Chronic renal disease**	53 (11.1)	9 (31)	<0.01
**Immunosuppression**	80 (16.7)	7 (24.1)	0.30
**Diabetes**	91 (19.1) *Miss = 2*	6 (20.7)	0.83
**Immunomodulatory treatment**	14 (4.3) *Miss = 156*	2 (8.3) *Miss = 5*	0.37
Characteristics on D1-D2			
**SAPS II**	36 [29;45] *Miss = 65*	43 [34;52]	0.02
**SOFA**	5 [4;6] *Miss = 156*	5 [5;6.5] *Miss = 6*	0.10
**Worst PaO2/FiO2[b]**	100 [72.2 ; 150]	80 [61 ; 122]	0.05
**IMV**	115 (24)	13 (44.9)	0.01
**Non IMV**	35 (7.4) *Miss = 4*	1 (3.4)	0.43
**HFNC**	229 (48.3) *Miss = 5*	14 (48.3)	1.00
**RRT**	17 (3.7) *Miss = 14*	4 (13.8)	<0.01
**Vasopressors**	105 (22.9) *Miss = 20*	12 (44.4) *Miss =2*	0.01
**Corticosteroids**	284 (59.5)	23 (79.3)	0.03
During ICU stay			
**Vasopressor**	159 (33.5) *Miss = 4*	27 (93.1)	<0.01
**IMV**	166 (34.8) *Miss = 2*	26 (89.7)	<0.01
**ECMO**	6 (1.5) *Miss = 69*	1 (3.8) *Miss = 3*	0.35
**RRT**	66 (13.9) *Miss = 3*	18 (62.1)	<0.01
**Corticosteroids**	314 (74.6) *Miss = 58*	25 (89.3)	0.08
**Tocilizumab**	34 (7.3) *Miss = 12*	4 (13.8)	0.20
**HSV[c]**	10 (2.1)	10 (34.5)	<0.01
**CMV[c]**	5 (1)	6 (20.7)	<0.01

**ICU LOS**[d]	7 [4;11]	19 [13;29]	<0.01
**ICU Death**	106 (22.1)	17 (58.6)	<0.01
**Hospital LOS**[d]	13 [9;22]<br>*Miss = 42*	20 [15;55]	<0.01
**Hospital death**	174 (36.3)	20 (69)	<0.01

Results are presented as median [IC] or value (%). a: years; b: mmHg, c: positive PCR on ≥1 blood sample; d: days. BMI: Body mass index, CV: cardiovascular, ECMO Extracorporeal membrane oxygenation, HFNC: High flow nasal cannula, ICU: Intensive care unit, IMV: Invasive mechanical ventilation, LOS: Length of stay, RRT: Renal replacement therapy, SAPS II: Simplified acute physiology score, SOFA: Sequential organ failure assessment

i. Subgroup analysis of ps/pb-CAPA and no-CAPA: laboratory results

	**Non CAPA (n=479)**	**Ps/pb CAPA (n=29)**	**P value**
Laboratory features on ICU admission	Median [IC]		
**Neutrophils**[a]	7000 [5000 ; 9960] *Miss = 82*	9820 [6825 ; 14050] *Miss = 9*	0.03
**Lymphocytes**[a]	795 [500 ; 1100] *Miss = 83*	650 [300 ; 900] *Miss = 9*	0.04
**Neutrophils/lymphocytes**	9.3 [5.7 ; 14.4] Miss=83	16.7 [9.1 ; 30.4] Miss=9	<.01
**Platelets**[a]	223 [169.5 ; 295] *Miss = 35*	235 [154 ; 288] *Miss = 8*	0.96
**D-dimers**[b]	1290 [800 ; 2500] *Miss = 82*	1300 [750 ; 1928] *Miss = 10*	0.77
**IL-10**	3 [1.5 ; 5.9] *Miss = 111*	6.1 [3.5 ; 9.4] *Miss = 2*	<.01
**IL-6**	15 [6 ; 31.6] *Miss = 26*	24.6 [14.1 ; 52.9]	0.02
**TNF- α**	1 [0 ; 2] *Miss = 35*	0.2 [0 ; 2.4] *Miss = 1*	0.55
**mHLA DR**[d]	10182 [7003 ; 14354] *Miss = 188*	6633.4 [5586 ; 10819] *Miss = 7*	0.01
**Il-6/mHLA-DR**	1.4 [0.5 ; 3.7]	3.2 [2.3 ; 4.5]	<0.01
Laboratory features after one week			
**IL-10**	2.1 [1.1 ; 4.1] *Miss = 259*	3.2 [1.9 ; 7] *Miss = 5*	0.03
**IL-6**	14 [5.7 ; 40] *Miss = 190*	48.1 [22 ; 122.3] *Miss = 4*	<0.01
**TNF- α**	0.6 [0 ; 1.8] *Miss = 263*	0.6 [0 ; 1.4] *Miss = 5*	0.78
**mHLA DR**[d]	8130.5 [5052 ; 13958.5] *Miss = 291*	5166 [3719.3 ; 5959] *Miss = 8*	<0.01
**Il-6/mHLA-DR**	1.7 [0.5 ; 7.9] *Miss = 292*	9 [3.1 ; 24.5] *Miss = 8*	<0.01
**ΔIL-10**[e]	-0.3 [-0.7 ; 0.1] *Miss = 265*	-0.2 [-0.7 ; 0] *Miss = 5*	0.92
**ΔIL-6** [e*]	0 [-0.6 ; 1.8] *Miss = 194*	0.3 [-0.1 ; 3] *Miss = 4*	0.06
**ΔmHLA DR**[e]	-0.1 [-0.5 ; 0.2] *Miss = 291*	-0.36 [-0.64; 0] *Miss = 8*	0.14
**ΔDelta IL-6/mHLA DR**[e]	0 [-0.8 ; 2.5] *Miss = 292*	1.2 [0.2 ; 5.2] *Miss = 8*	0.03

a: G/L, b: ng/mL, c: pg/L, d: mHLA-DR count on monocyte, e: (Day 7 value minus Day 1 Value) of Day 1 value. HLA: Human leukocyte antigen, Il: Interleukin, TNF: Tumor necrosis factor. Serum levels of cytokines were previously standardized as described in the method section.

j. Subgroup analysis of ps/pb-CAPA and s-CAPA populations

	**Ps/pb-CAPA**	**s-CAPA**	**p-value**
**Number of patients**	29	18	.
**Time from symptoms to admission**	9 [6 ; 11]	7.5 [6 ; 11]	0.38
**Time from hospital to ICU admission**	1 [1 ; 5]	2 [1 ; 4]	0.54
**Age**[a]	72 [66 ; 76]	67.4 [60.5 ; 72.2]	0.06
**Gender (Male)**	20 (69)	15 (83.3)	0.27
**BMI > 30 kg/m²**	13 (44.8)	9 (50)	0.73
**Main comorbidities**			
**Chronic CV disease**	7 (24.1)	6 (33.3)	0.49
**Chronic respiratory disease**	5 (17.2)	3 (16.7)	0.96
**Chronic renal disease**	9 (31)	2 (11.1)	0.12
**Immunosuppression**	7 (24.1)	3 (16.7)	0.54
**Diabetes**	6 (20.7)	4 (22.2)	0.90
Characteristics on D1-D2			
**SAPS II**	43 [34 ; 52]	37.5 [27 ; 42]	0.06
**SOFA**	5 [5 ; 7]	5 [5 ; 7]	0.62
**PaO2/FiO2**[b]	80 [61 ; 122]	76.3 [60 ; 97.1]	0.45
**IMV**	13 (44.8)	2 (11.1)	0.02
**Non IMV**	1 (3.4)	2 (11.1)	0.30
**HFNC**	14 (48.3)	15 (83.3)	0.02
**RRT**	4 (13.8)	0 (0)	0.10
**Vasopressors**	12 (44.4)	2 (11.1)	0.02
**Corticosteroids**	23 (79.3)	13 (72.2)	0.58
During ICU stay			
**Vasopressors**	27 (93.1)	16 (88.9)	0.61
**IMV**	26 (89.7)	16 (88.9)	0.93
**ECMO**	1 (3.8)	1 (5.6)	0.79
**RRT**	18 (62.1)	7 (38.9)	0.12
**Corticosteroids**	25 (89.3)	17 (94.4)	0.54
**Tocilizumab**	4 (13.8)	2 (11.1)	0.79
**HSV**[c]	10 (34.5)	6 (33.3)	0.94
**CMV**[c]	6 (20.7)	2 (11.1)	0.40
**ICU LOS**[d]	19 [13 ; 29]	26 [17 ; 46]	0.27
**ICU Death**	18 (62.1)	12 (66.7)	0.75
**Hospital LOS**	20 [15 ; 55]	34.5 [18 ; 61]	0.11
**Hospital Death**	20 (69)	12 (66.7)	0.87

Results are presented as median [IC] or value (%). a: years; b: mmHg, c: positive PCR on ≥1 blood sample; d: days. BMI: Body mass index, CV: cardiovascular, ECMO Extracorporeal membrane oxygenation, HFNC: High flow nasal cannula, ICU: Intensive care unit, IMV: Invasive mechanical ventilation, LOS: Length of stay, RRT: Renal replacement therapy, SAPS II: Simplified acute physiology score, SOFA: Sequential organ failure assessment

k. Subgroup analysis of ps/pb-CAPA and s-CAPA: laboratory results

	**Ps/pb-CAPA**	**s-CAPA**	
Number of patients	29	18	.
Laboratory features on ICU admission			
**Neutrophils**[a]	9820 [6825 ; 14050]<br>Miss = 9	8025 [6900 ; 11750]<br>Miss = 1	0.68
**Lymphocytes**[a]	650 [300 ; 900]<br>Miss= 9	550 [400 ; 800]<br>Miss = 1	0.95
**Platelets**[a]	235 [154 ; 288]<br>Miss=8	215 [158 ; 285]<br>Miss = 1	0.86
**D-dimers**[b]	1300 [800 ; 4100]<br>Miss=10	1300 [900 ; 1600]<br>Miss = 1	0.89
**IL-10**	6.1 [3.5 ; 9.4]<br>Miss=2	3.1 [2.3 ; 7]<br>Miss = 2	0.19
**IL-6**	24.6 [14.1 ; 52.9]	22.2 [11.2 ; 46]<br>Miss = 1	0.67
**TNF- α** *	0.2 [0 ; 2.4]<br>Miss = 1	0 [0 ; 1.7]<br>Miss = 2	0.70
**mHLA-DR**[d]	6633.4 [5586 ; 10819]<br>Miss = 7	6645 [3887 ; 13536]<br>Miss = 3	0.89
**Il-6/mHLA-DR**	3.2 [2.3 ; 4.5]<br>Miss = 7	3 [1.1 ; 8.2]<br>Miss = 3	0.70
Laboratory features after one week			
**IL-10**	3.2 [1.9 ; 7]<br>Miss=5	2.9 [1.8 ; 3.7]<br>Miss = 5	0.38
**IL-6**	48.1 [22 ; 122.3]<br>Miss=4	44.5 [13.1 ; 105.8]<br>Miss = 4	0.76
**TNF- α**	0.6 [0 ; 1.4]<br>Miss=5	0.5 [0 ; 1.5]<br>Miss = 5	0.64
**mHLA DR**[d]	5166 [3719.3 ; 5959]<br>Miss=8	5092 [3433 ; 12509]<br>Miss = 8	0.84
**Il-6/mHLA-DR**	9 [3.1 ; 24.5]<br>Miss=8	8.4 [1.3 ; 24.4]<br>Miss = 8	0.53
**ΔIL-10**[e]	-0.2 [-0.7 ; 0]<br>Miss=5	-0.4 [-0.6 ; 0]<br>Miss = 5	0.99
**ΔIL-6** [e]	0.3 [-0.1 ; 3]<br>Miss=4	0.8 [-0.6 ; 4]<br>Miss = 4	0.66
**ΔmHLA-DR** [e]	-0.4 [-0.6 ; 0]<br>Miss=8	-0.3 [-0.7 ; 0]<br>Miss = 10	0.93
**ΔIL-6/mHLA DR**[e]	1.2 [0.2 ; 5.2]<br>Miss=8	0 [-0.4 ; 2]<br>Miss = 11	0.24

a: G/L, b: ng/mL, c: pg/L, d: mHLA-DR count on monocyte, e: (Day 7 value minus Day 1 Value) of Day 1 value. HLA: Human leukocyte antigen, Il: Interleukin, TNF: Tumor necrosis factor. Serum levels of cytokines were previously standardized as described in the method section.

l. Analysis of ps/pb-CAPA and matched no-CAPA (M2no-CAPA) populations

	Matched No-CAPA (N=52)	Ps/pb-CAPA (N=28)	P-val
**Time from symptoms to admission**	9 [5 ; 13]	9 [6 ; 11]	0.55
**Time from hospital to ICU admission**	2 [1 ; 5]	1 [1 ; 5]	0.63
**Age[a]**	71 [65.7 ; 78.1]	72 [66 ; 76]	0.81
**Gender (Male)**	36 (69.2)	20 (69)	0.98
**BMI > 30 kg/m²**	21 (40.4)	13 (44.8)	0.70
**Main comorbidities**			
**Chronic CV disease**	10 (19.2)	7 (24.1)	0.60
**Chronic respiratory disease**	2 (3.8)	5 (17.2)	0.04
**Chronic renal disease**	7 (13.5)	9 (31)	0.06
**Immunosuppression**	8 (15.4)	7 (24.1)	0.33
**Diabetes**	9 (17.3)	6 (20.7)	0.71
Characteristics on D1-D2			
**SAPS II**	41 [33 ; 50] *Miss = 1*	43 [34 ; 52]	0.75
**SOFA**	7 [5 ; 10] *Miss = 24*	5 [5 ; 7]	0.26
**PaO2/FiO2[b]**	84.1 [72.4 ; 117.2]	80 [61 ; 122]	0.50
**IMV**	22 (42.3)	13 (44.8)	0.83
**Non IMV**	3 (5.8)	1 (3.4)	0.64
**HFNC**	24 (46.2)	14 (48.3)	0.85
**RRT**	4 (8) *Miss = 2*	4 (13.8)	0.41
**Vasopressors**	16 (32) *Miss = 2*	12 (44.4) *Miss = 2*	0.28
**Corticosteroids**	43 (82.7)	23 (79.3)	0.71
During ICU stay			
**Vasopressors**	27 (51.9)	27 (93.1)	<0.01
**IMV**	29 (55.8)	26 (89.7)	<0.01
**ECMO**	0 (0) *Miss = 5*	1 (3.8) *Miss = 3*	0.18
**RRT**	12 (23.1)	18 (62.1)	<0.01
**Corticosteroids**	46 (95.8) *Miss = 4*	25 (89.3)	0.27
**Tocilizumab**	3 (5.8)	4 (13.8)	0.22
**HSV[c]**	3 (5.8)	10 (34.5)	<0.01
**CMV[c]**	1 (1.9)	6 (20.7)	<0.01
**ICU LOS[d]**	10 [8 ; 15.5]	19 [13 ; 29]	<0.01
**ICU Death**	23 (44.2)	18 (62.1)	0.12
**Hospital LOS**	17 [13 ; 24] *Miss = 1*	20 [15 ; 55]	0.07
**Hospital Death**	27 (51.9)	20 (69)	0.14

Results are presented as median [IC] or value (%). a: years; b: mmHg, c: positive PCR on ≥1 blood sample; d: days. BMI: Body mass index, CMV: Cytomegalovirus, CV: cardiovascular, ECMO Extracorporeal membrane oxygenation, HFNC: High flow nasal cannula, HSV: Herpes simplex virus, ICU: Intensive care unit, IMV: Invasive mechanical ventilation, LOS: Length of stay, RRT: Renal replacement therapy, SAPS II: Simplified acute physiology score, SOFA: Sequential organ failure assessment

m. Analysis of ps/pb-CAPA and matched no-CAPA (M2no-CAPA): laboratory results

	Matched cohort (N=52)	Ps/pb CAPA (N=28)	pvalue
Laboratory features on ICU admission			
**Neutrophils**[a]	7162.5 [4315 ; 10716.7]<br>*Miss = 4*	9820 [6825 ; 14050]<br>*Miss = 9*	0.12
**Lymphocytes**[a]	600 [500 ; 950]<br>*Miss = 4*	650 [300 ; 900]<br>*Miss = 9*	0.52
**Platelets**[a]	203 [162 ; 267]<br>*Miss = 1*	235 [154 ; 288]<br>*Miss = 8*	0.42
**D-dimers**[b]	1200 [1000 ; 2800]<br>*Miss = 6*	1300 [800 ; 4100]<br>*Miss = 10*	0.82
**IL-10**	2.8 [1.5 ; 6.4]<br>*Miss = 5*	6.1 [3.5 ; 9.4]<br>*Miss = 2*	0.01
**IL-6**	13.3 [6.8 ; 25.7]<br>*Miss = 2*	24.6 [14.1 ; 52.9]	<0.01
**TNF- α**	1.9 [1.2 ; 2.6]<br>*Miss = 3*	0.2 [0 ; 2.4]<br>*Miss = 1*	<0.01
**mHLA DR**[d]	9392 [7347 ; 11647]<br>*Miss = 14*	6633.4 [5586 ; 10819]<br>*Miss = 7*	0.15
**Il6/mHLA DR**	2.2 [1 ; 3.8]<br>*Miss = 15*	3.2 [2.3 ; 4.5]<br>*Miss = 7*	0.05
Laboratory features after one week			
**IL-10**	1.9 [1 ; 4.1]<br>*Miss = 12*	3.2 [1.9 ; 7]<br>*Miss = 5*	0.03
**IL-6**	12.5 [5.8 ; 26.1]<br>*Miss = 9*	48.1 [22 ; 122.3]	<0.01
**TNF- α**	1.2 [0 ; 2.4]<br>*Miss = 12*	0.6 [0 ; 1.4]<br>*Miss = 5*	0.2
**mHLA DR**[d]	7658.6 [4988 ; 11837]<br>*Miss = 16*	5166 [3719 ; 5959]<br>*Miss = 8*	<0.01
**IL-6/HLA DR**	1.7 [0.4 ; 5.9]<br>*Miss = 11*	9 [3.1 ; 24.5]<br>*Miss = 8*	<.01
**ΔIL-10**[e]	-0.3 [-0.7 ; 0]<br>*Miss = 14*	-0.2 [-0.7 ; 0]<br>*Miss = 5*	0.96
**ΔIL-6**[e]	-0.1 [-0.6 ; 1.1]<br>*Miss = 9*	0.3 [-0.1 ; 3]<br>*Miss = 3*	0.03
**ΔmHLA DR**[e]	0 [-0.4 ; 0.7]<br>*Miss = 16*	-0.4 [-0.6 ; 0]<br>*Miss = 8*	0.09
**ΔIL-6/mHLA DR**[e]	0.1 [-0.6 ; 2.6]<br>*Miss = 16*	1.2 [0.2 ; 5.2]<br>*Miss = 8*	0.18

a: G/L, b: ng/mL, c: pg/L, d: HLA-DR count on monocyte, e: (Day 7 value minus Day 1 Value) of Day 1 value. HLA: Human leukocyte antigen, Il: Interleukin, TNF: Tumor necrosis factor, Serum levels of cytokines were previously standardized as described in the method section.

n. Risk factor of ps/pb CAPA in the ps/pb CAPA + matched cohort M2no-CAPA population– conditional logistic regression

Variable	OR	OR IC	pvalue
**Gender (male)**	1.08	[0.42 ; 2.78]	0.87
**BMI > 30 kg/m²**	1.17	[0.46 ; 3.00]	0.74
**Chronic CV Disease**	1.37	[0.46 ; 4.05]	0.58
**Chronic respiratory failure**	7.71	[0.88 ; 67.7]	0.07
**CKD**	2.99	[0.87 ; 10.19]	0.08
**Diabetes**	1.17	[0.40 ; 3.44]	0.78
**RRT**	1.69	[0.42 ; 6.85]	0.46
**Vasopressors**	4.90	[0.57 ; 42.41]	0.14
**Corticosteroids**			
**Tocilizumab**	4.65	[0.46 ; 46.88]	0.19
**Neutrophils > 8 G/L** [a]	2.93	[1.08 ; 7.98]	0.04
**Lymphocytes > 0.6 G/L** [a]	1.45	[0.60 ; 3.54]	0.41
**D-dimers > 1500 ng/mL** [a]	0.95	[0.33 ; 2.78]	0.93
**PCT >0.5 µg/L** [a]	1.27	[0.40 ; 4.08]	0.69
**IL-10 > 3.2** [a]	2.27	[0.837 ; 6.16]	0.11
**IL-6 > 15.8** [a]	2.00	[0.729; 5.49]	0.17
**TNF- α > 1** [a]	0.66	[0.217; 2.03]	0.47
**HLA DR > 8000** [a]	0.41	[0.17; 0.99]	0.042
**IL-6/mHLA-DR > 2.8** [a]	1.94	[0.83; 4.55]	0.13
**ΔIL-6 > 0**[b]	1.69	[0.58; 4.90]	0.346
**ΔHLA DR>0**[b]	0.22	[0.05; 1.10]	0.07
**ΔIL-6/HLA DR>0**[b]	3.54	[0.96; 13.1]	0.06

a: Value on D1, b: (Day 7 value minus Day 1 Value) of Day 1 value. BMI: Body mass index, CKD: Chronic kidney disease, CV: Cardiovascular, HLA: Human leukocyte antigen, IL: Interleukin, IMV: Invasive mechanical ventilation, mHLA-DR: HLA-DR count on monocyte, RRT: Renal replacement therapy, PCT: Procalcitonin. Serum levels of cytokines were previously standardized as described in the method section.

o. Risk factor of ps/pb CAPA in the ps/pb CAPA + matched cohort M2no-CAPA population– conditional logistic regression (multivariate analysis)

	aOR	[CI 95%]	p-value
**CKD**	3.97	[1.05; 14.97]	0.04
**Neutrophils > 8 G/L** [a]	2.95	[1.03; 8.44]	0.04

a: Value on D1, aOR: adjusted odds ratio, CKD : Chronic kidney disease

p. Risk factor of ps/pb CAPA or death in the ps/pb CAPA + matched cohort M2no-CAPA population– conditional logistic regression

Variable	OR	OR IC	p-value
**Gender (male)**	2	[0.64;6.24]	0.23
**BMI > 30 kg/m²**	0.78	[0.27;2.26]	0.65
**Chronic CV Disease**	1.21	[0.28;5.2]	0.8
**Chronic respiratory failure**	9.1	[1.06;78.44]	0.04
**CKD**	3.94	[0.74;20.89]	0.11
**Diabetes**	1.1	[0.26;4.71]	0.9
**IMV**	1.1	[0.26;4.71]	0.9
**RRT**	1.69	[0.23;12.23]	0.61
**Remdesivir**	1.36	[0.2;9]	0.75
**Tocilizumab**	4	[0.36;44.11]	0.26
**Neutrophils > 8 G/L**[a]	2.66	[0.85;8.27]	0.09
**Lymphocytes > 0.6 G/L**[a]	0.84	[0.27;2.63]	0.77
**Neutrophils/Lymphocytes >10**[a]	1.98	[0.62; 6.32]	0.25
**D-dimers > 1500 ng/mL**[a]	3.1	[0.63;15.38]	0.17
**PCT >0.5 µg/L**[a]	3.14	[0.34;28.58]	0.31
**IL-10 > 3.2 pg/L**[a]	4.08	[1.09;15.36]	0.04
**IL-1 > 0.4 pg/L**[a]	0.49	[0.12;2.02]	0.32
**IL-6 > 15.8 pg/L**[a]	3.49	[0.94;13.05]	0.06
**TNF- α > 1 pg/L**[a]	1.94	[0.52;7.25]	0.32
**mHLA-DR > 8000 antibodies/cell**[a]	0.26	[0.08;0.81]	0.02
**IL-6/mHLA-DR > 2.8**[a]	3.16	[0.98;10.21]	0.05
**ΔIL-10 > 0**[b]	0.57	[0.18;1.82]	0.34
**ΔIL-6 > 0**[b]	2.03	[0.58;7.14]	0.27
**ΔmHLA-DR > 0**[b]	3.71	[0.97;14.27]	0.06

a: D1 value, b: (Day 7 value minus Day 1 Value) of Day 1 value. BMI: Body mass index, CKD: Chronic kidney disease, CV: Cardiovascular, HLA: Human leukocyte antigen, IL: Interleukin, IMV: Invasive mechanical ventilation, RRT: Renal replacement therapy, PCT: Procalcitonin. Serum levels of cytokines were previously standardized as described in the method section.

q. Flow chart of the main article

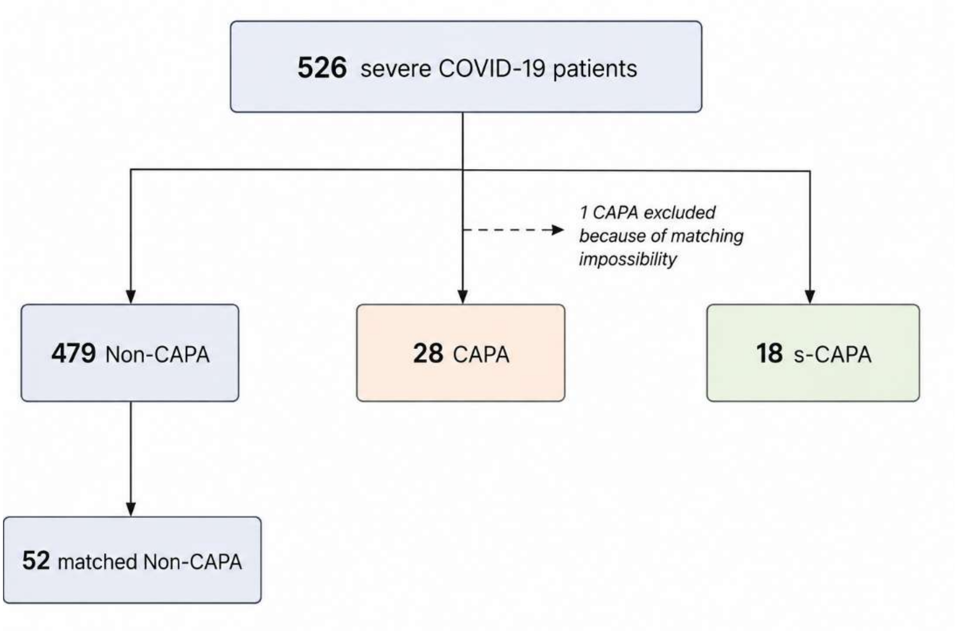

CAPA: Covid-19-associated pulmonary aspergillosis, ICU: Intensive care unit

r. Flow chart of the whole cohort

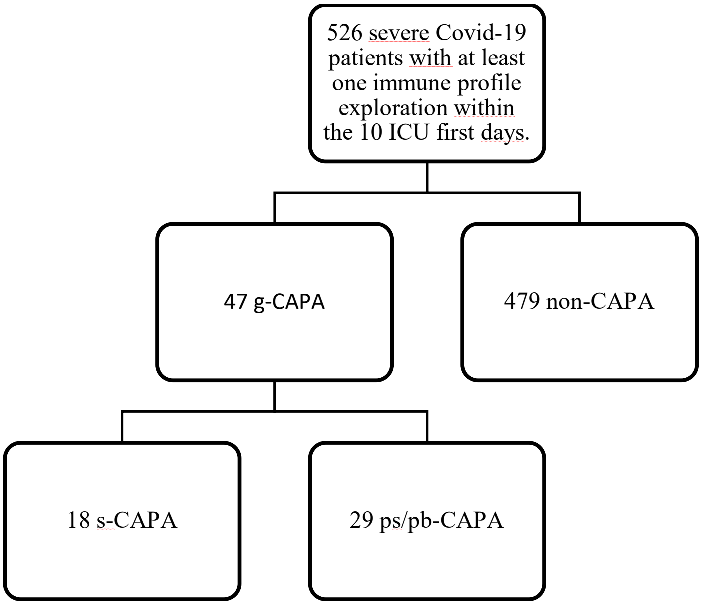

CAPA: Covid-19 associated pulmonary aspergillosis, g-CAPA: gathered CAPA, ICU: Intensive care unit, ps/pb-CAPA: possible or probable CAPA, s-CAPA: suspected CAPA

s. Boxplot of the inflammatory biomarkers depending on the timing of the dosage and CAPA in the cohort of 47 g-CAPA versus matched no-CAPA cases (M1no-CAPA)

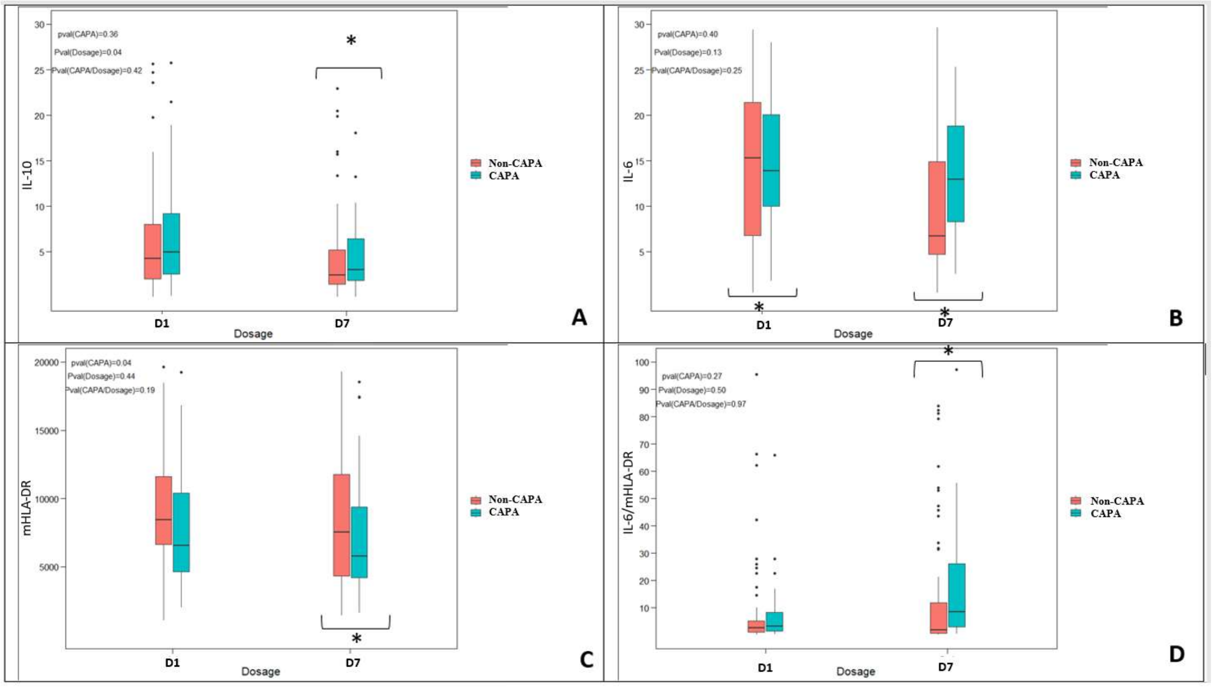

Boxplots representing levels of Interleukin 10 (A), Interleukin 6 (B), mHLA-DR expression (C) and IL-6/mHLA-DR ratio (D). Red for non-CAPA patients and blue for g-CAPA patients; Interleukin levels are schematized in pg/L, mHLA-DR is schematized in count of HLA-DR on monocyte surface, *:p<0.05, 1st : Day 1 sample, 2nd : Day 7 sample, IL : interleukin, HLA: mHLA-DR. Serum levels of cytokines were previously standardized as described in the method section.

t. Boxplot of the inflammatory biomarkers depending on the timing of the dosage and CAPA in the cohort of 29 ps/pb CAPA versus 52 matched cases (M2no-CAPA)

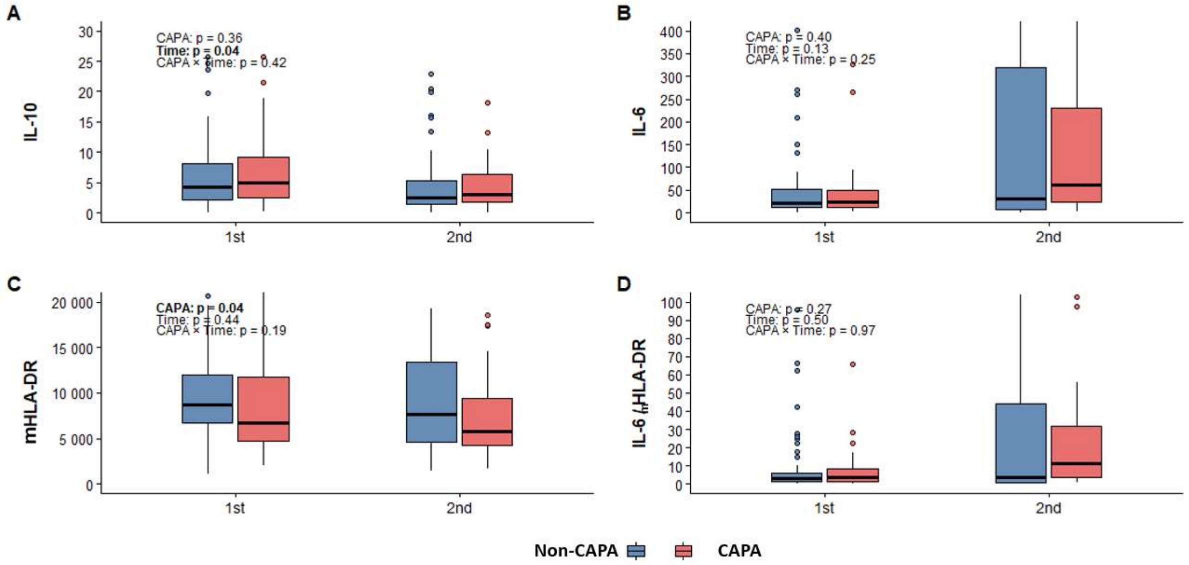

Boxplots representing levels of Interleukin 10 (A), Interleukin 6 (B), mHLA-DR expression (C) and IL-6/mHLA-DR ratio (D); Interleukin levels are schematized in pg/L, mHLA-DR is schematized in count of HLA-DR on monocyte surface, *:p<0.05, 1st : Day 1 sample, 2nd : Day 7 sample, IL : interleukin, HLA: mHLA-DR. Serum levels of cytokines were previously standardized as described in the method section.

u. Biological diagnosis data

Possible and probable CAPA

**Probable CAPA biological criteria**	**n = 22**	**Possible CAPA biological criteria**	**n = 7**
Positive microscopy of *aspergillus* spp on BAL	3 (14)	Positive microscopy of *aspergillus* spp on tracheal aspirate	5 (71)
Positive *aspergillus* spp culture on BAL	7 (32)	Positive *aspergillus* spp culture on tracheal aspirate	6 (86)
Blood galactomannan index > 0.5	12 (55)	Positive *aspergillus* spp PCR on tracheal aspirate	1 (14)
BAL galactomannan index > 1.0	3 (14)		
Aspergillus spp PCR positive with Ct<36	8 (36)		
ICU LOS before first positive sample for ps/pb CAPA[a]	8 [7.19; 14.97]<br>*Miss = 3*		

Results are presented as median [IC] or value (%). a: in days. BAL: Bronchoalveolar lavage, CAPA: COVID-19 associated pulmonary aspergillosis, Ct: Cycle threshold, ICU: Intensive care unit, LOS: Length of stay, PCR: Polymerase chain reaction, ps/pb CAPA: Possible or probable CAPA.

Suspected CAPA

**Suspected CAPA criteria**	**n = 18**	**BDG value[a]**
Aspergillus spp PCR on tracheal aspirate with Ct = 36	1 (6)	
Aspergillus spp culture positive on sputum culture	1 (6)	
BDG 30 pg/mL and treatment received	17 (94)	140.4 [111.31; 268.01]

Results are presented as median [IC] or value (%). a: in pg/mL. BAL: Bronchoalveolar lavage, BDG: beta-D-glucan, CAPA: COVID-19 associated pulmonary aspergillosis, Ct: Cycle threshold, PCR: Polymerase chain reaction. Treatment: voriconazole or isavuconazole or liposomal amphotericin B

v. Description of the main population according by center

	**All cohort**				**Patients with CAPA**			
	Clermont Ferrand	LYON	PARIS	P-value	Clermont Ferrand	LYON	PARIS	P-value
	N=366	N=67	N=94	.	N=24	N=3	N=2	.
**Time from symptoms to admission**	9 [6 ; 12]	7 [4 ; 10]	8 [5 ; 12]	0.020	9 [6.5 ; 10.5]	14 [4 ; 19]	8 [6 ; 10]	0.702
**Time from hospital to ICU admission**	2 [1 ; 5]	0 [0 ; 1]	0 [0 ; 1]	<.001	1.5 [1 ; 5]	0 [0 ; 2]	3.5 [0 ; 7]	0.185
**Age[a]**	68.4 [59.99 ; 75.12]	65 [52 ; 72]	58.66 [53.34 ; 66.57]	<.001	74.01 [67.82 ; 78.18]	58 [52 ; 62]	63.43 [58.23 ; 68.63]	0.011
**Gender (male)**	250 (68.306)	53 (79.104)	67 (71.277)	0.200	15 (62.5)	3 (100)	2 (100)	0.257
**BMI > 30 kg/m²**	157 (42.896)	23 (34.328)	23 (24.468)	0.004	10 (41.667)	3 (100)		0.067
**Main comorbidities**								
**Chronic CV disease**	74 (20.219)	2 (2.985)	14 (15.217)	0.002	7 (29.167)			0.382
**Chronic respiratory disease**	34 (9.29)	6 (8.955)	16 (17.391)	0.071	5 (20.833)			0.533
**Chronic renal disease**	33 (9.016)	5 (7.463)	27 (28.723)	<.001	5 (20.833)	2 (66.667)	2 (100)	0.025
**Immunosuppression**	61 (16.667)	4 (5.97)	25 (26.596)	0.003	5 (20.833)	1 (33.333)	1 (50)	0.603
**Diabetes**	57 (15.574)	17 (25.373)	27 (29.348)	0.004	6 (25)			0.455
Characteristics on D1-D2								
**SAPS II**	36 [29 ; 45]	35 [27 ; 45]	43 [31 ; 55]	0.175	41 [30.5 ; 50]	44 [36 ; 55]	52 [50 ; 54]	0.365
**PaO2/FiO2[b]**	93 [64 ; 130.95]	139 [90 ; 191]	106 [84 ; 156]	<.001	73 [57.5 ; 126.5]	107 [95 ; 267]	91.5 [64 ; 119]	0.402
**IMV**	50 (13.661)	39 (58.209)	41 (43.617)	<.001	9 (37.5)	2 (66.667)	2 (100)	0.169
**Non IMV**	34 (9.29)		4 (4.444)	0.014			1 (50)	<.001
**HFNC**	218 (59.563)	15 (23.077)	31 (34.066)	<.001	12 (50)	1 (33.333)	1 (50)	0.861
**RRT**	18 (4.918)	2 (2.985)	1 (1.25)	0.288	3 (12.5)		1 (50)	0.257
**Vasopressors**	51 (13.934)	24 (48.98)	44 (48.889)	<.001	9 (37.5)	1 (100)	2 (100)	0.121
**Corticosteroids**	289 (78.962)	2 (2.985)	31 (32.979)	<.001	21 (87.5)		2 (100)	0.002
During ICU stay								
**Vasopressors**	117 (31.967)	41 (61.194)	46 (51.111)	<.001	22 (91.667)	3 (100)	2 (100)	0.800
**IMV**	118 (32.24)	41 (61.194)	50 (54.348)	<.001	21 (87.5)	3 (100)	2 (100)	0.706
**ECMO**	4 (1.093)		4 (4.494)	0.029	1 (4.167)			0.768
**RRT**	63 (17.213)	15 (22.388)	13 (14.286)	0.410	14 (58.333)	2 (66.667)	2 (100)	0.499
**Corticosteroids**	309 (99.038)	18 (26.866)	30 (33.708)	<.001	21 (91.304)	2 (66.667)	2 (100)	0.379
**Tocilizumab**	35 (9.563)		5 (6.098)	0.022	4 (16.667)			0.617
**ICU LOS[d]**	7 [4 ; 13]	10 [3 ; 30]	7 [4 ; 9]	0.023	20.5 [12.5 ; 29]	52 [18 ; 53]	12.5 [11 ; 14]	0.157
**ICU Death**	102 (27.869)	18 (26.866)	15 (15.957)	0.060	16 (66.667)	1 (33.333)	1 (50)	0.499
**Hospital LOS**	14 [9 ; 24]	21 [11 ; 56]	12 [8 ; 16.5]	<.001	21 [14.5 ; 49]	71 [20 ; 72]	16 [14 ; 18]	0.191
**Hospital Death**	127 (34.699)	23 (34.328)	57 (60.638)	<.001	18 (75)	1 (33.333)	1 (50)	0.283

Results are presented as median [IC] or value (%). a: years; b: mmHg, c: positive PCR on ≥1 blood sample; d: days. BMI: body mass index, CV: cardiovascular, ECMO extracorporeal membrane oxygenation, HFNC: high-flow nasal cannula, ICU: Intensive Care Unit, IMV: invasive mechanical ventilation, LOS: length of stay, n: number, RRT: renal replacement therapy; SAPS II: simplified acute physiology score, SOFA: sequential organ failure assessment. Immunosuppression: Hematologic malignancy (active or in remission for less than 5 years), hematopoietic stem cell transplant for less than 5 years, active solid cancer, leukopenia < 1 G/L or neutropenia ≤ 0.5 G/L, solid organ transplantation, syndrome acquired immunodeficiency, long-term corticosteroid therapy ≥ 0.5 mg/kg/day of prednisone equivalent for at least 3 weeks, immunosuppressive or immunomodulatory treatment

w. Description of CAPA patients according to FUNDICU guidelines and matched Non-CAPA patients

		Matched No CAPA	FUNDICU-CAPA	Miss	pvalue
		56 (100)	19 (100)	0	.
**Participating center**	Cl_Fer	18 (32.1)	17 (89.5)	0	<.01
	HCL	38 (67.9)	0 (0)	.	.
	Paris	0 (0)	2 (10.5)	.	.
**Time from symptom onset to ICU admission, days, median (IQR)**		7 [4 ; 10]	10 [7 ; 12]	1	0.02
**Time from hospital admission to ICU admission, days, median (IQR)**		0.5 [0 ; 2]	1 [1 ; 5]	0	<.01
**Age, years, median (IQR)**		70.2 [66 ; 76]	73 [66.4 ; 76]	0	0.17
**Body mass index (BMI), kg/m^2, median (IQR)**		27.1 [24.6 ; 30.6]	28.2 [24.2 ; 32.5]	1	0.32
**Male sex, n (%)**		45 (80.4)	10 (52.6)	0	0.02
**Obesity, n (%)**		18 (32.1)	8 (42.1)	0	0.43
**Current or former smoking, n (%)**		3 (16.7)	2 (10.5)	38	0.59
**Chronic liver disease, n (%)**		4 (7.1)	0 (0)	2	0.26
**Cardiovascular disease, n (%)**		7 (12.5)	5 (26.3)	0	0.16
**Chronic respiratory disease, n (%)**		9 (16.1)	1 (5.3)	0	0.23
**Chronic kidney disease, n (%)**		4 (7.1)	3 (15.8)	0	0.26
**Immunosuppression, n (%)**		6 (10.7)	3 (15.8)	0	0.56
**Diabetes mellitus, n (%)**		14 (25)	4 (21.1)	0	0.73
**Charlson Comorbidity Index, median (IQR)**		2 [1 ; 5]	2 [0 ; 3]	40	0.28
**Previous immunosuppressive therapy, n (%)**		1 (5.6)	1 (5.9)	40	0.97
**SAPS II score, median (IQR)**		37 [30 ; 45.5]	43 [29 ; 52]	0	0.11
**Respiratory SOFA score (Day 1–2)**		19 (95)	14 (77.8)	37	0.12
**Coagulation SOFA score (Day 1–2)**				37	.
**Cardiovascular SOFA score (Day 1–2)**		4 (20)	8 (44.4)	37	0.11
**Neurological SOFA score (Day 1–2)**		1 (5)	1 (5.6)	37	0.94
**Hepatic SOFA score (Day 1–2)**				37	.
**Renal SOFA score (Day 1–2)**		0 (0)	3 (16.7)	37	0.06
**Total SOFA score, median (IQR)**		5.5 [5 ; 8]	5.5 [5 ; 7]	37	0.43
**PaO_2/FiO_2 ratio, median (IQR)**		96.7 [76.3 ; 150]	77 [55 ; 122]	2	0.06
**Invasive mechanical ventilation, n (%)**		26 (46.4)	9 (47.4)	0	0.94
**Non-invasive ventilation, n (%)**		2 (3.6)	0 (0)	0	0.4
**HNC, n (%)**		27 (50)	10 (52.6)	2	0.84
**Extracorporeal membrane oxygenation (ECMO), n (%)**				38	.
**Renal replacement therapy (RRT), n (%)**		2 (3.6)	3 (15.8)	0	0.07
**Vasopressor therapy, n (%)**		17 (37)	9 (47.4)	10	0.44
**Corticosteroid therapy, n (%)**		15 (26.8)	17 (89.5)	0	<.01
**Anakinra therapy, n (%)**				40	.
**Hydroxychloroquine therapy, n (%)**		18 (32.1)	0 (0)	0	<.01
**Remdesivir therapy, n (%)**		2 (3.6)	0 (0)	0	0.4
**Lopinavir/ritonavir therapy, n (%)**		6 (10.7)	1 (5.3)	0	0.48
**Vasopressor therapy during ICU stay, n (%)**		40 (71.4)	17 (89.5)	0	0.11
**Mechanical ventilation during ICU stay, n (%)**		41 (73.2)	16 (84.2)	0	0.33
**ECMO during ICU stay, n (%)**		1 (5.6)	1 (5.3)	38	0.97

**Renal replacement therapy during ICU stay, n (%)**		14 (25)	10 (52.6)	0	0.03
**Corticosteroid therapy during ICU stay, n (%)**		29 (51.8)	17 (89.5)	0	<.01
**Therapeutic plasma exchange, n (%)**		1 (1.8)	0 (0)	0	0.56
**Tocilizumab therapy, n (%)**		2 (3.6)	3 (15.8)	0	0.07
**Herpes simplex virus infection, n (%)**		7 (12.5)	8 (42.1)	0	<.01
***Aspergillus* infection, n (%)**		2 (11.1)	12 (70.6)	40	<.01
**Cytomegalovirus infection, n (%)**		2 (3.6)	3 (15.8)	0	0.07
**CMV reactivation, n (%) *(si c'est bien cela)***		2 (3.6)	3 (15.8)	0	0.07
**Fungemia, n (%)**		1 (1.8)	1 (6.7)	5	0.32
**Liposomal amphotericin B treatment, n (%)**				39	.
**Isavuconazole treatment, n (%)**		14 (77.8)	9 (50)	39	0.08
**Voriconazole treatment, n (%)**		3 (16.7)	7 (38.9)	39	0.14
**Leukocyte count ($\times 10^9$/L), median (IQR)**		8710 [7000 ; 11440]	12000 [6000 ; 18000]	14	0.05
**Neutrophil count ($\times 10^9$/L), median (IQR)**		7335 [4800 ; 9200]	11400 [8800 ; 16100]	16	<.01
**Lymphocyte count ($\times 10^9$/L), median (IQR)**		790 [500 ; 1100]	800 [600 ; 1000]	16	0.42
**Platelet count ($\times 10^9$/L), median (IQR)**		186 [138 ; 251]	261 [201 ; 335]	9	<.01
**Ferritin (µg/L), median (IQR)**		918 [600 ; 1708]	1680 [1196 ; 2674]	43	0.03
**C-reactive protein (CRP), mg/L, median (IQR)**		141.4 [94.4 ; 201]	150.3 [43.3 ; 304]	45	0.5
**D-dimer, median (IQR)**		1266 [800 ; 1600]	1664 [750 ; 5600]	34	0.13
**Procalcitonin, median (IQR)**		0.3 [0.1 ; 0.9]	1.1 [0.3 ; 3.4]	39	0.02
**Interleukin-10 (Day 1), median (IQR)**		3.1 [2.3 ; 7]	6.4 [3.5 ; 9.4]	41	0.13
**Interleukin-6 (Day 1), median (IQR)**		22.2 [11.2 ; 46]	41.3 [18.1 ; 71.4]	41	0.06
**TNF-α (Day 1), median (IQR)**		0 [0 ; 1.7]	0.1 [0 ; 0.4]	41	0.36
**Monocyte HLA-DR expression (Day 1), median (IQR)**		10333 [6645 ; 14688]	6500 [4962 ; 9335]	11	0.05
**IL-6/HLA-DR ratio (Day 1), median (IQR)**		3 [1.1 ; 8.2]	4.3 [2.8 ; 10.5]	48	0.13
**Interleukin-10 (Day 7), median (IQR)**		2.9 [1.8 ; 3.7]	3 [1.9 ; 7.7]	45	0.28
**Interleukin-6 (Day 7), median (IQR)**		44.5 [13.1 ; 105.8]	53.9 [42.1 ; 147.3]	45	0.18
**TNF-α (Day 7), median (IQR)**		0.5 [0 ; 1.5]	0.4 [0 ; 1.4]	45	0.47
**Monocyte HLA-DR expression (Day 7), median (IQR)**		6279 [4380 ; 8983]	5166 [3506 ; 5734]	25	0.05
**IL-6/HLA-DR ratio (Day 7), median (IQR)**		8.4 [1.3 ; 24.4]	14.4 [5.5 ; 26.1]	51	0.11
**Change in IL-10 (Day 7 – Day 1), median (IQR)**		-0.4 [-0.6 ; 0]	-0.3 [-0.7 ; 0]	45	0.31
**Change in IL-6 (Day 7 – Day 1), median (IQR)**		0.8 [-0.6 ; 4]	0.1 [-0.4 ; 4.5]	45	0.43
**Change in TNF-α (Day 7 – Day 1), median (IQR)**		-0.3 [-0.9 ; 0.2]	0 [-0.3 ; 2.7]	59	0.21
**Change in HLA-DR expression (Day 7 – Day 1), median (IQR)**		-0.3 [-0.6 ; 0.1]	-0.3 [-0.5 ; 0]	29	0.5
**Change in IL-6/HLA-DR ratio (Day 7 – Day 1), median (IQR)**		0 [-0.4 ; 2]	0.5 [0 ; 15.5]	54	0.22
**Hospital length of stay, days, median (IQR)**		30.5 [16.5 ; 60]	19 [14 ; 38]	0	0.05
**ICU length of stay, days, median (IQR)**		19 [7.5 ; 43]	15 [11 ; 26]	0	0.32
**Time to death, days, median_**		26 [17 ; 34.5]	16 [12 ; 26]	49	0.03
**ICU death, n(%)**		12 (21.4)	12 (63.2)	0	<.01
**Hospital death, n(%)**		28 (50)	14 (73.7)	0	0.07

x. Risk factor of FUNDICU CAPA in the FUNDICU CAPA and matched cohort – Univariate conditionnal logictic regression

Variable	Odds ratio (95% CI)	P value
**Patient characteristics**		
Age (per year)	1.27 (0.48–3.39)	0.627
Male sex	0.31 (0.09–1.05)	0.061
Obesity	2.56 (0.72–9.12)	0.147
**Comorbidities**		
Cardiovascular disease	<0.001 (0–999.99)	0.999
Chronic respiratory disease	0.33 (0.04–2.70)	0.309
Chronic kidney disease	3.00 (0.50–17.95)	0.229
Immunosuppression	6.00 (0.40–89.91)	0.34
Diabetes mellitus	0.76 (0.21–2.74)	0.671
HIV infection	0.50 (0.16–1.61)	0.246
**ICU therapies**		
Renal replacement therapy	3.00 (0.55–16.13)	0.248
Catecholamine therapy (Day 1–2)	0.95 (0.30–2.99)	0.924
Corticosteroid therapy (Day 1–2)	<0.001 (0–999.99)	0.997
Hydroxychloroquine	<0.001 (0–999.99)	0.994
Remdesivir	<0.001 (0–999.99)	0.996
Lopinavir/ritonavir	0.35 (0.04–3.35)	0.36
Interferon treatment	<0.001 (0–999.99)	0.995
**Immunological biomarkers**		
IL-10	0.50 (0.15–1.67)	0.261
IL-6	0.50 (0.14–1.84)	0.293
TNF-α	<0.001 (0–999.99)	0.996
HLA-DR expression	0.45 (0.14–1.44)	0.179
IL-6/HLA-DR ratio	2.10 (0.52–8.43)	0.295
ΔIL-10	1.16 (0.31–4.31)	0.823
ΔIL-6	1.32 (0.47–3.69)	0.597
ΔHLA-DR	0.50 (0.09–2.78)	0.429
ΔIL-6/HLA-DR ratio	2.61 (0.76–8.96)	0.127
